# Specialized adaptation of a lactic acid bacterium to the milk environment: the comparative genomics of *Streptococcus thermophilus* LMD-9

**DOI:** 10.1186/1475-2859-10-S1-S22

**Published:** 2011-08-30

**Authors:** Yong Jun Goh, Caitlin Goin, Sarah O’Flaherty, Eric Altermann, Robert Hutkins

**Affiliations:** 1 Department of Food, Bioprocessing and Nutrition Sciences, North Carolina State University, Raleigh, North Carolina 27695, USA; 2 School of Biological Sciences, University of Nebraska—Lincoln, Lincoln, Nebraska 68588, USA; 3 AgResearch Limited, Grasslands Research Centre, Palmerston North, New Zealand; 4 Riddet Institute, hosted by Massey University, Palmerston North, New Zealand; 5 Department of Food Science and Technology, University of Nebraska—Lincoln, Lincoln, Nebraska 68583, USA

## Abstract

**Background:**

*Streptococcus thermophilus* represents the only species among the streptococci that has “Generally Regarded As Safe” status and that plays an economically important role in the fermentation of yogurt and cheeses. We conducted comparative genome analysis of *S. thermophilus* LMD-9 to identify unique gene features as well as features that contribute to its adaptation to the dairy environment. In addition, we investigated the transcriptome response of LMD-9 during growth in milk in the presence of *Lactobacillus delbrueckii* ssp. *bulgaricus*, a companion culture in yogurt fermentation, and during lytic bacteriophage infection.

**Results:**

The *S. thermophilus* LMD-9 genome is comprised of a 1.8 Mbp circular chromosome (39.1% GC; 1,834 predicted open reading frames) and two small cryptic plasmids. Genome comparison with the previously sequenced LMG 18311 and CNRZ1066 strains revealed 114 kb of LMD-9 specific chromosomal region, including genes that encode for histidine biosynthetic pathway, a cell surface proteinase, various host defense mechanisms and a phage remnant. Interestingly, also unique to LMD-9 are genes encoding for a putative mucus-binding protein, a peptide transporter, and exopolysaccharide biosynthetic proteins that have close orthologs in human intestinal microorganisms. LMD-9 harbors a large number of pseudogenes (13% of ORFeome), indicating that like LMG 18311 and CNRZ1066, LMD-9 has also undergone major reductive evolution, with the loss of carbohydrate metabolic genes and virulence genes found in their streptococcal counterparts. Functional genome distribution analysis of ORFeomes among streptococci showed that all three *S. thermophilus* strains formed a distinct functional cluster, further establishing their specialized adaptation to the nutrient-rich milk niche. An upregulation of CRISPR1 expression in LMD-9 during lytic bacteriophage DT1 infection suggests its protective role against phage invasion. When co-cultured with *L. bulgaricus*, LMD-9 overexpressed genes involved in amino acid transport and metabolism as well as DNA replication.

**Conclusions:**

The genome of *S. thermophilus* LMD-9 is shaped by its domestication in the dairy environment, with gene features that conferred rapid growth in milk, stress response mechanisms and host defense systems that are relevant to its industrial applications. The presence of a unique exopolysaccharide gene cluster and cell surface protein orthologs commonly associated with probiotic functionality revealed potential probiotic applications of LMD-9.

## Background

Originally described by Orla-Jensen in 1919 [1], *Streptococcus thermophilus* is a low G + C, Gram-positive, nonmotile, non-spore-forming, catalase-negative, facultative anaerobic, homofermentative lactic acid bacterium that has restricted natural habitats in the bovine mammary mucosa and raw milk. Among the ninety-three currently classified species from the genus *Streptococcus*[2], *S. thermophilus* represents the only species that is “Generally Regarded As Safe” (GRAS). Accordingly, *S. thermophilus* plays a prominent role in food biopreservation. Notably, the species is used extensively in yogurt, cheese, and other dairy fermentations, where it is traditionally paired with *Lactobacillus delbrueckii* subsp. *bulgaricus* or *Lactobacillus helveticus*. In these products, *S. thermophilus* is responsible for rapid acidification as well as formation of the expected flavor and texture properties [3]. In addition, yogurt cultures have also been linked to various probiotic effects, including the alleviation of lactose intolerance [4], modulation of intestinal microbiota [5], immunostimulation of host production of pro-inflammatory and anti-inflammatory cytokines [6,7], and inhibition of specific periodontal pathogens [8].

There are currently 48 completed genome sequences from the genus *Streptococcus* that are listed in the National Center for Biotechnology Information (NCBI) Microbial Genomes database. In addition, 311 streptococcal genome sequencing projects are currently in progress. Among these, three complete genomes belong to *S. thermophilus*, with a fourth that is in progress. The genome blueprints of *S. thermophilus* LMD-9 and two other *S. thermophilus* strains, CNRZ1066 and LMG 18311 [9], have led to the identification of several important features. Not surprisingly, genes associated with virulence in other streptococci are either non-functional or completely absent in *S. thermophilus*[9,10], suggesting that reductive evolution had occurred during its domestication in the nutritionally-rich milk niche. In addition, the LMD-9 genome contains regions unique to this strain, including genes encoding for the production and regulation of a broad spectrum bacteriocin, Thermophilin 9, genes involved in quorum sensing and competence development [11-16], and several of the clustered regularly interspaced short palindromic repeats (CRISPR) regions not present in both CNRZ1066 and LMG 18311 that are involved in bacteriophage defense [17,18].

Interestingly, long before even the notion of a genome had even been conceived, *S. thermophilus* was reportedly known “more by the things which it cannot do than by its positive actions" [19]. Numerous studies have led to the realization that this view is no longer accurate and that *S. thermophilus* is indeed genetically equipped to perform numerous biological functions [20]. In this current report, we describe the many unique properties present in this organism, as revealed by comparative genomic analyses of LMD-9 with other *S. thermophilus* strains as well as commensal and pathogenic streptococcal species. We also present results obtained from transcriptome analysis and in-depth mining of the annotated LMD-9 genome to uncover other genetic features that are potentially relevant to its adaptation and performance in the dairy environment.

## Methods

### Genome sequencing

The genome sequencing of *Streptococcus thermophilus* LMD-9 (ATCC BAA-491) was described previously [10]. The complete genome sequence and curated annotation of *S. thermophilus* LMD-9 can be accessed at GenBank under accession number CP000419 (chromosome), CP000420 (cryptic plasmid pSTER_A) and CP000421 (cryptic plasmid pSTER_B).

### Bioinformatic analyses

The GAMOLA [21]/Artemis [22] software suite was used to manage the genome annotation. Protein-encoding open reading frames (ORFs) were identified using the ORF-prediction program Glimmer2 [23]. ORFeome previously predicted by Makarova *et al*. [10] was used as a reference. A manual inspection was performed in order to verify or, if necessary, redefine the start and stop positions of each ORF based on sequence alignment with proteins in the non-redundant protein database provided by NCBI and potential ribosomal binding sites. Assignment of protein function to ORFs was performed manually using results from several sources. BLASTP [24] was use on both a non-redundant protein database provided by NCBI and clusters of orthologous groups (COG) [25] databases. HMMER [26] was used to identify protein motifs to the PFAM libraries [27]. TMHMM [28] was used to predict transmembrane sequences, and SignalP [29] was used for the prediction of signal peptides. The TatP prediction tool was used to search for proteins that contain a TAT signal peptide and TAT motif [30]. Chromosomal and plasmid maps were constructed using the Microbial Genome Viewer [31] and Clone Manager Professional software (Sci-Ed Software, Cary, NC), respectively.

Whole genome sequences were aligned using the GenomeComp software tool [32] with the external MEGABLAST nucleotide sequence alignment search program available from NCBI (ftp://ftp.ncbi.nih.gov/blast/) and default run parameters. Metabolic pathway mapping of *S. thermophilus* LMD-9 ORFeome was executed using the software suite PathwayVoyager [33] with the Kyoto Encyclopedia of Genes and Genomes (KEGG) online database (http://www.genome.jp/kegg/kegg2.html).

A Functional Genome Distribution analysis (FGD, E. Altermann, in preparation) was performed using 39 publicly available *Streptococcus* genome sequences, including *S. thermophilus* LMD-9 (Additional file 1). FGD analyzes the functional similarity between microbes based on their predicted ORFeomes. FGD is a comparative genomics approach to genome-genome comparisons, emphasizing functional relationships rather than ancestral lineages. Briefly, pooled ORFeomes are subjected to all-versus-all analyses, evaluating the level and quality of amino-acid similarities between individual ORFs pairings. Results for each genome-genome combination are then combined into a symmetrical distance matrix and was visualized using the Unweighted Pair Group Method with Arithmetic mean (UPGMA) method [34]. Strain and cluster conserved and specific gene sets were mined based on respective BLAST e-values, using custom developed software. The distance matrix was imported into MEGA4 [35] for visualization. Based on the FGD calculations, ORFeome synteny on amino acid level between all genomes was assessed using ACT [36].

### Transcriptome analysis. (i) Bacteriophage infection

*S. thermophilus* LMD-9 was incubated in M17 + glucose at 42°C until reaching OD625 ~ 0.4, then the culture was split. The lytic bacteriophage DT1 was added at a M.O.I. of 1 to one culture as the expression treatment and samples were taken at time points of 5 min before infection and 0, 5, 10, 20, 30, and 40 min after infection. The cells of the other half of the culture were immediately harvested by centrifugation as the control.

### (ii) *S. thermophilus* and *L. bulgaricus* synergism in milk

*L. bulgaricus* BAA-365 was grown in skim milk for 7 hrs at 42°C, and then added at an equal volume to *S. thermophilus* LMD-9, also in skim milk. Sterile skim milk at a similar pH was added to a parallel culture of LMD-9 as a control. After 4 hours or when pH 5.5 was reached, cells were collected for RNA isolation.

### (iii) Microarray fabrication, hybridization and data analysis

Microarrays were fabricated as 60mer oligo-chip arrays generated from the *S. thermophilus* LMD-9 genome (Invitrogen, Carlsbad, CA). Each oligomer was contact-printed using the OminGrid robotic arrayer (GeneMachine, San Carlos, CA), in triplicate, for a total of 4,866 features per microarray. Slides were pre-treated according to the manufacturer’s recommendations using a UV cross-linking method to anchor the oligos to the surface of the epoxy slide.

Cells grown under various growth conditions were harvested by centrifugation, and RNAprotect (Qiagen Inc., Valencia, CA) was used to stop gene expression and stabilize the RNA [37]. RNA isolation was achieved using the chaotropic agent TRI reagent (Molecular Research Center, Inc., Cincinnati, OH) according to the manufacturer’s instructions, followed by bead-beating and chloroform extraction, purification, and DNase treatment (Turbo DNase, Ambion/Applied Biosystems, Austin, TX). The cDNA was synthesized using Superscript II Reverse Transcriptase (Invitrogen) from 30 μg of extracted RNA and directly labeled with two different fluorochromes (Perkin Elmer Inc., Waltham, MA); Cy3 was used to label the treatment group and Cy5 was used to label the control group. The labeled probes were hybridized to the microarray slides in a HybChamber (GeneMachine) for 16 to 20 hours at 43 °C, then the slides were washed in a series of three washing buffers [(1) 1X SSC, 0.03% SDS, (2) 0.2x SSC, and (3) 0.05 x SSC] and scanned using a GenePix 4000B scanner (Axon Instruments/Molecular Devices, Inc., Sunnyvale, CA) at 5 μm per pixel resolution.

Statistical analysis of the data was based on the median pixel intensities (at wavelengths of 635 and 532 nm) generated by the GenePix scanner. The values were normalized between spots and between each of the three replicates performed using LimmaGui software package (http://bioinf.wehi.edu.au/limmaGUI/) with general loess after background correction. The least squares method was used to determine differentially expressed genes, and only those genes with a p value of ≤ 0.05 were considered significantly different.

## Results and discussion

### General genome features

**Figure 1:**
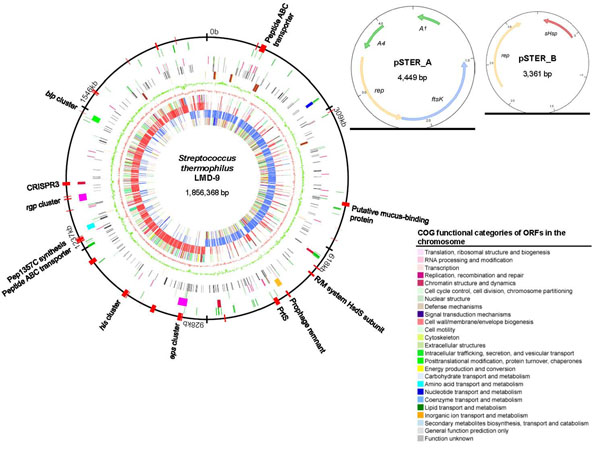
**Genome maps of the *S. thermophilus* LMD-9 chromosome and two cryptic plasmids, pSTER_A and pSTER_B.** From the innermost towards the outermost circles of the chromosome map: Circle 1, COG functional classification of ORFs on the forward strand; Circle 2 (blue), ORFs on the forward strand; Circle 3 (red), ORFs on the reverse strand; Circle 4, COG functional classification of ORFs on the reverse strand; Circle 5 (salmon), GC skew; Circle 6 (green), GC composition (%); Circle 7, rRNAs and tRNAs, shown in brown and orange, respectively; Circle 8 (dark grey), pseudogenes; Circle 9, intact and truncated transposases, shown in red and green, respectively; Circle 10, key features encoded in the chromosome – urease gene cluster (blue), CRISPR regions (dark red), prophage remnant (yellow), *eps* and *rgp* clusters (fuchsia), *gal-lac* cluster (cyan) and *blp* cluster (green); Circle 11 (green), *S. thermophilus-*specific genes not present in *S. salivarius* SK126 draft genome; Circle 12 (red), LMD-9 specific genes or chromosomal regions not present in both CNRZ1066 and LMG 18311. Selected features within the unique regions are indicated. Genes encoding the Pep1357C cyclic peptide was previously identified by Ibrahim et al. [15]. Color designation for COG classification of ORFs in Circle 1 and 4 is shown at the bottom right legend.

The general genome features of *S. thermophilus* LMD-9 was previously described as part of a multiple lactic acid bacteria genomes sequencing project and comparative analysis [10]. The LMD-9 genome is comprised of a single circular chromosome (1,856,368 bp; 39.1% G + C) and two cryptic plasmids, pSTER_A (4,449 bp; 37.0% G + C) and pSTER_B (3,361 bp; 35.1% G + C) (Figure 1). Collectively, the LMD-9 genome is approximately 60 kb larger than the genomes of the previously sequenced plasmid-free strains LMG 18311 and CNRZ1066 [9] (Table 1). Despite the modest differences in genome size, all three genomes have the same G + C content of 39.1%. The LMD-9 chromosome contains 1,834 predicted open reading frames (ORFs) that accounts for 81.2% of the chromosomal sequence. The ORFeome has an overall G + C content of 39.93% and an average gene length of 822 bp. Putative biological roles have been assigned for 1,417 (77%) of the ORFs, whereas the remaining 417 ORFs encode for hypothetical proteins in which no probable function could be predicted. As many as 241 (13%) ORFs appear to be pseudogenes (Additional file 2). The majority of these pseudogenes encode for transposases, hypothetical proteins, carbohydrate transport and metabolism, and transcriptional regulators.

**Table 1:** General genome features of *S. thermophilus*

	*S. thermophilus* strain		
Feature	LMD-9	CNRZ1066	LMG 18311
	Danisco	Yogurt isolate (France)	Yogurt isolate (UK)
Origin of strain	(USA)	(France)	(UK)
Size of chromosome (bp)	1,856,368	1,796,226	1,796,846
G + C content (%)	39.08	39.08	39.09
No. of predicted ORFs	1,834	1,915	1,890
ORFs with putative functions	1,417 (77%)	1,372 (71.6%)	1,376 (72.8%)
Coding density	81%	84%	84%
Pseudogenes (% ORFs)	241 (13.1%)	182 (9.5%)	180 (9.5%)
No. of rRNA operons	6	6	6
No. of tRNAs	67	67	67
Prophage remnant	1	1	0
Plasmid	2	0	0
Genbank Accession no.	CP000419	CP000024	CP000023
Reference	10, this study	9	9

### Genome comparison with *S. thermophilus* LMG 18311 and CNRZ1066

**Figure 2:**
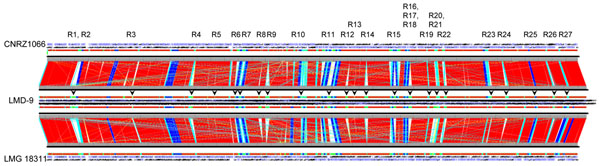
****Alignment of *S. thermophilus* genomes.**** Genome alignment of *S. thermophilus* LMD-9 with CNRZ1066 and LMG 18311 strains was performed using the GenomeComp software tool [32]. Alignment lengths are color-coded as follows: red, >10,000 bp; blue, 5,000 to 10,000 bp; cyan, 1,000 to 5,000 bp; yellow, 500 to 1,000 bp, and pink, <500 bp. Unique regions are in green. LMD-9 specific regions are indicated with black arrowheads above the LMD-9 linear chromosome, with region designation shown at the top of the upper canvas. ORFs present within these regions are described in Additional file 3.

Approximately 83% of the *S. thermophilus* LMD-9 ORFeome are common to both CNRZ1066 and LMG 18311 and constitutes the backbone of these three strains. The latter two strains shared about 93 to 94% of their ORFs [9]. As observed previously [38], the LMD-9 genome exhibited a lesser degree of global synteny when compared to both LMG 18311 and CNRZ1066, although all three genomes are essentially collinear (Figure 2).

### (i) LMD-9 specific regions

Genome alignment revealed chromosomal regions of approximately 137 kb (7.4%) and 144 kb (10.6%) in the LMD-9 strain that are not present in CNRZ1066 and LMG 18311, respectively. These genomic segments correspond mainly to 73 and 65 regions of >50 bp specific in LMD-9 when compared to CNRZ1066 and LMG 18311 genomes, respectively. Collectively, an approximate 114 kb of LMD-9 chromosomal regions encompassing 127 ORFs (representing 6.9% of the LMD-9 ORFeome) are not found in both LMG 18311 and CNRZ1066 (Additional file 3). These ORFs are located in 27 chromosomal loci, six of which spanned more than 8 kb in length (Figure 2). Interestingly, the distribution of these 27 loci appears to concentrate at regions distal from the origin of DNA replication (Figure 1, circle 12). Remarkably, 14 of these 27 LMD-9 specific chromosomal loci are flanked by intact or truncated transposases, suggesting IS elements as a significant contributor to the genome diversity in *S. thermophilus*.

Deduced proteins from 57 (45%) of the 127 LMD-9 specific ORFs have no orthologous counterpart in other streptococcal species (see below), indicating they likely originated via lateral gene transfer (LGT). Approximately one-fifth (24/127) of the LMD-9 specific ORFs are predicted pseudogenes, providing further evidence that gene decay had continued following strain divergence. The majority of the remaining intact genes (40/103 or 39%) encode for proteins involved in amino acid transport and metabolism, transposases, or proteins having unknown functions.

Eight of the LMD-9 specific regions contain ORFs with conserved homologs in *Streptococcus salivarius* (more than 90% sequence identity), notably genes coding for glycosyltranferases (R12 and R20), the *his* gene cluster (R14), and numerous unknown proteins, suggesting that these ORFs were most likely lost in both LMG 18311 and CNRZ1066 strains. Similar to both LMG 18311 and CNRZ1066, genes implicated in virulence from the pathogenic streptococci are either absent or truncated in the LMD-9 genome, or are involved in other physiological functions relevant to the adaptation towards dairy environment. In addition, only 17 of the LMD-9 specific ORFs have closest orthologs with the pathogenic streptococci. Most of these genes encode for unknown proteins and transporters, some of which are truncated. One region (R9) encoding a partial spermidine/putrescine ABC transporter, along with a putative chloride channel and a cell envelope serine proteinase PrtS, is flanked by transposases and showed conserved sequence and synteny to *Streptococcus suis*[*39*]. While the absence of PrtS in both LMG 18311 and CNRZ1066 strains has been previously noted [16], this region, a so-called “ecological island”, was frequently found in the chromosomes of recently emerged *S. thermophilus* industrial strains [39].

Three regions (R2, R5, and R11) encode for proteins related to exopolysaccharides (EPS) biosynthesis with closest identities to corresponding proteins from *Enterococcus faecalis* (STER0149, STER0150), *Lactobacillus rhamnosus* (STER0656), *Lactobacillus gasseri* (STER1061), *Lactococcus lactis* (STER1057), *Clostridium beijerinckii* (STER1059), and intriguingly, gastrointestinal-specific bacteria *Eubacterium rectale* and *Ruminococcus obeum* (STER1063-1065). Aside from the aforementioned PrtS, LMD-9 also acquired an additional dipeptide/oligopeptide ABC transporter (STER0142-STER0145) located within R2 that potentially contributes to nutrient uptake in milk environments. This peptide transporter exhibited moderate similarity (50 to 60% identity) to the corresponding transporter components from various *Bifidobacterium* species also specifically associated with the human gut. Eight other LMD-9 specific regions encode for proteins that potentially broadened its repertoire of defense mechanisms. These included two copies of a putative antimicrobial peptide ABC transporter (STERψ0571-0572 and STER1347-1348) that have close orthologs in *Leuconostoc mesenteroides* and *Lactobacillus fermentum*. Also identified were components of restriction-modification (R/M) systems (STER0731 and STER0750), an abortive infection phage resistance protein (STER1698), and the previously identified CRISPR3 region (R22) [18] which showed moderate sequence homology (60 to 87% identity) to orthologs in *Streptococcus mutans*, *Streptococcus agalactiae*, *Streptococcus dysgalactiae*, *Streptococcus equi*, and *Streptococcus pyogenes*. Strain LMD-9 also encodes a 1,009-amino acid residues surface protein in R4 (STER0576) that was closely related to mucus-binding (MucBP) domain-containing proteins in lactobacilli. This putative mucus-binding protein (Mub) is not found in other streptococci, and has a single MucBP domain with an N-terminal YSIRK signal peptide and a C-terminal LPNTG cell wall anchoring motif.

The genome of LMD-9 harbors a prophage remnant (STER0810-0830) where part of the region (STER0816-0830) showed high degree of conservation with the prophage remnant in CNRZ1066. Examination of the corresponding chromosomal region in LMG 18311 and *S. salivarius* SK126 draft genome, both of which lack the phage remnant, indicated that the prophage elements in both LMD-9 and CNRZ1066 were integrated between the genes encoding for para-aminobenzoate synthases component I (STER0809/str0771) and 5-methyltetrahydropteroyltriglutamate--homocysteine S-methyltransferase (STER0831/str0785). The remaining 3.7 kb of the prophage region (R8), which is present only in LMD-9, exhibited a high degree of sequence homology with lactococcal phages, suggesting the acquisition of this region after strain divergence.

### (ii) Regions absent in LMD-9

Approximately 105 kb and 103 kb in total of chromosomal regions in CNRZ1066 and LMG 18311, respectively, were absent in the LMD-9 genome. Overall, 57 ORFs interspersed in 14 chromosomal regions were present in both strains but not in LMD-9. Fifteen of these ORFs are pseudogenes, including remnants of gene clusters coding for an amidase (str/stu0973), an amino acid ABC transporter substrate-binding protein (str/stu0975), and a maltose/maltodextrin ABC transporter (str/stu1015-strstu1017). These genes appear intact in *S. salivarius* and *S. mutans*, indicating that like LMD-9, these genes are also undergoing degeneration, albeit at a slower rate, in the other two strains. On the other hand, as in *Streptococcus gordonii*, both CNRZ1066 and LMG 18311 possessed a second copy of biotin synthase-encoding gene *bioY2* (str/stu1308) in a 8.6-kb unique region (str/stu1308-1315), which displayed 42% sequence identity to *bioY1* present in all three *S. thermophilus* strains. The remainder of the region encodes for a proline/glycine ABC transporter that showed 60-70% similarity to the corresponding orthologs in *Streptococcus gallolyticus*, and a partial L-histidine degradation pathway consisting of histidine ammonia-lyase (*hutH*, str/stu1313; EC 4.3.1.3) and a urocanate hydratase (*hutU*, str/stu1315; EC 4.2.1.49). These enzymes are involved in the initial steps of the pathway, although both CNRZ1066 and LMG 18311 lack imidazolonepropionase (EC 3.5.2.7) and formiminoglutamase (EC 3.5.3.8) required to yield the final product, L-glutamate. Both strains also appeared to have acquired type III R/M system components (str/stu 0884 and 0885) that most closely resembled the orthologs in *Listeria monocytogenes* (70 to 80% identity). Strain LMD-9 also lacks genes encoding two oxidative stress defense proteins (str/stu0183 and str/stu1314) in CNRZ1066 and LMG 18311 that are homologous (99% identity) to the OsrE and OsrF of *S. thermophilus* CNRZ368 [40].

### Genome comparison of *S. thermophilus* with other streptococci

**Figure 3:**
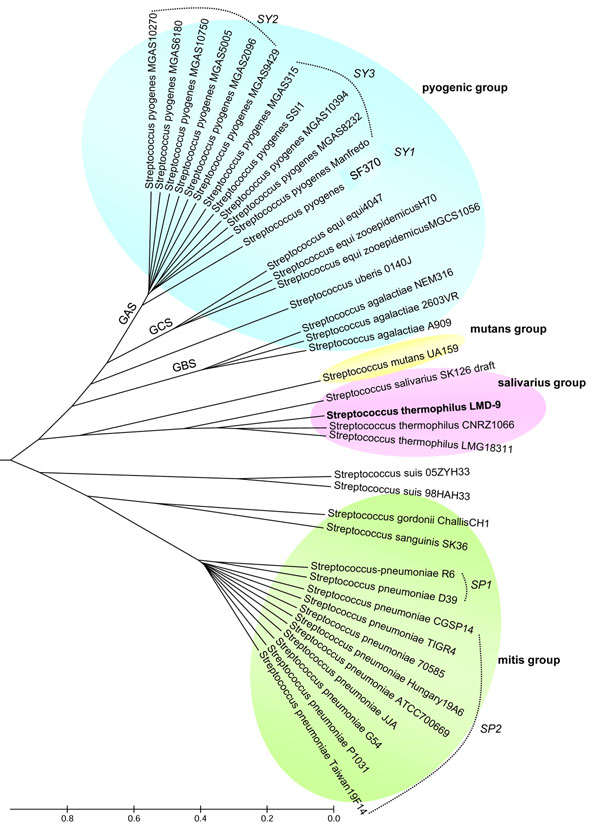
****Functional genome distribution (FGD) tree of 39 *Streptococcus* genomes.**** FGD analysis of 39 *Streptococcus* strains for which genome sequences are available, depicting the functional relationship among the species and strains based on the amino acid sequence similarities of the predicted ORFeomes. *S. thermophilus* strains formed a functional cluster with *S. salivarius* as the closest relative. The FGD analysis also predicted functional sub-clusterings among the *S. pneumoniae* and *S. pyogenes* strains. Approximated branch lengths, representing the level of functional similarity between genomes are depicted by distance units (du).

Comparative analysis of the ORFeomes among sequenced streptococci using the FGD analysis revealed functional clustering of the species that is consistent with their grouping (pyogenic, mutans, salivarius, and mitis) based on 16S rDNA phylogenetic inferences [41] (Figure 3). Both *S. thermophilus* CNRZ1066 and LMG 18311 formed a tight cluster that diverges from the LMD-9 strain. The *S. thermophilus* strains and the other member of the salivarius group, *S. salivarius* SK126 (a human skin isolate), formed a functional cluster, with *S. mutans* as the closest pathogenic relative. Comparison of genome content between all three *S. thermophilus* strains and the draft genome of *S. salivarius* SK126 revealed that both species shared approximately 75% of the protein-coding genes (E-value cutoff 1e-10). A set of 68 genes was identified as unique for *S. thermophilus* compared to SK126, including genes encoding urease biogenesis, lactose utilization (lactose permease LacS and beta-galactosidase LacZ), CRISPR elements, transposases, various transporters, a type II R/M system, and numerous hypothetical proteins of unknown function (Additional file 4). It is interesting to note that the urease gene cluster, *lacS* and *lacZ* are commonly found in human oral-associated strains of *S. salivarius* but apparently are absent in the skin-associated SK126 strain. Since these genes are not universally present in other streptococci, it is plausible that they were acquired by clonal populations of *S. salivarius* and *S. thermophilus* via LGT in their natural habitats. Meanwhile, 428 genes from *S. salivarius* SK126 are absent in all three *S. thermophilus* strains. *S. salivarius* has a broader repertoire of genes involved in carbohydrate utilization and biosynthesis of complex polysaccharides, including an elaborate set of glucosyltransferases for glucan biosynthesis, as well as the complete pathway for the synthesis of glycogen (STRSA0001_0011 to STRSA0001_0014). In addition to the SrtA sortase, which is present as an intact gene only in LMD-9 among the sequenced *S. thermophilus* strains, *S. salivarius* also possesses an SrtB sortase, and a wider array of cell surface proteins.

Among all the sequenced streptococcal strains included in the comparative analysis, 510 genes were found conserved (E-value ≤ 1e-60 cutoff) in all strains. These core genes, which are mostly involved in housekeeping functions, account for 27.8% of the total ORFs encoded in the LMD-9 chromosome. Among the non-core genes, only 13 have no significant identity to all other streptococci and therefore are species-specific for *S. thermophilus*, which includes a putative arginase/agmatinase, a homoserine/threonine efflux protein, a probable serine/threonine protein kinase, oxidoreductases, transposases and conserved hypothetical proteins (Additional file 4). Meanwhile, 8 genes were identified as conserved in all streptococci excluding *S. thermophilus*, notably genes that encode for enzymes from the tagatose-6-phosphate pathway, a transaldolase (EC 2.2.1.2) involved in the pentose phosphate pathway, and a carbamate kinase (EC 2.7.2.2) belonging to the arginine deiminase pathway.

Recent extensive genome analyses of pathogenic streptococci have established that capsular polysaccharide genes and phage elements are major contributors to the genome diversity among strains of *Streptococcus pneumoniae*[42,43] and *S. pyogenes*[44-48], respectively. Our comparative genome analysis revealed functional sub-clusterings among the sequenced strains of *S. pneumoniae* and *S. pyogenes* (Figure 3). Within the *S. pneumoniae* strains, eleven genes were detected as unique for both R6 and D39 (cluster SP1), notably genes encoding for capsular polysaccharide biosynthesis (Additional file 5). On the other hand, the SP1 strains lack seven genes that are conserved in all cluster 2 (SP2) strains of *S. pneumoniae*. Aside from capsular polysaccharide biosynthetic genes, both clusters also differ by the exclusive presence of a bacteriocin immunity protein BlpX and a prophage maintenance system killer protein in the SP2 cluster strains. It was previously reported in the TIGR4 (an SP2 strain) genome that the bacteriocin biosynthesis and immunity protein genes are among those that underwent lineage-specific gene duplication [42]. Meanwhile, the *S. pyogenes* strains appeared as three subclusters, with an apparent divergence of the M1 strain SF370 (subcluster SY1) from the other strains, including the contemporary M1 strain MGAS5005. Only four genes, Spy0942, Spy0944, Spy0978 and Spy2147, were detected as SF370-specific, of which the latter three are phage-associated proteins (Additional file 6). Interestingly, no ORF was detected as conserved in all other *S. pyogenes* strains that is absent in SF370. The major difference between the remaining SY2 and SY3 subclusters is the presence of lantibiotic biosynthetic genes, phage-related genes, and virulence-associated genes, such as fibronectin-binding protein and serum opacity factor in the SY2 subcluster.

### Metabolic and biosynthetic capabilities

The LMD-9 genome encodes sets of enzymes conserved in the LMG 18311 and CNRZ1066 strains for glycolytic pathway, the non-oxidative branch of the pentose phosphate pathway, and pyruvate metabolism [16]. Unlike other closely-related Gram-positive species of lactococci, enterococci, bacilli, listeria and lactobacilli, the pyruvate carboxylase-encoding gene required for the formation of oxaloacetate from pyruvate is absent in all streptococci. Instead, the key precursor for the biosynthesis of aspartate family amino acids is generated from phosphoenolpyruvate by a phosphoenolpyruvate carboxylase (Ppc) conserved in the streptococci. In *S. thermophilus*, it was demonstrated that the activity of Ppc is essential for the bioavailability of L-aspartic acid during growth in milk [49].

Unlike its pathogenic counterparts such as *S. agalactiae*, *S. pyogenes* and *S. pneumoniae*, which are auxotrophic for most amino acids, the putative pathways for the biosynthesis of all amino acids with the possible exception of alanine and lysine were identified in the LMD-9 genome. The absence or inactivation of these enzymes will also affect peptidoglycan biosynthesis, which suggests that the generation of key intermediates (L-alanine and *meso*-diaminopimelate) for peptidoglycan synthesis may involve an alternate pathway. LMD-9 has a 7.4-kb *his* gene cluster (STER1198 to STER1207) that encodes a complete histidine biosynthetic pathway, but appears to be deleted in LMG 18311 and CNRZ1066 [16], which is further evidenced by the relative conservation of the adjacent genes flanking the *his* cluster between LMD-9 and *S. salivarius* SK126. Nonetheless, in LMD-9, we speculated that the presence of several copies of transposases at the upstream vicinity of the *his* gene loci might be involved in the inactivation of an EPS biosynthesis protein homolog (ψSTER1211) and the deletion of glycosyltransferase genes which are otherwise found intact in the corresponding region in *S. salivarius*.

*S. thermophilus* is predicted to be capable of *de novo* synthesis of both purine and pyrimidine nucleotides. Like the majority of the streptococci, *S. thermophilus* has the gene which encodes for nucleoside diphosphate kinase (EC 2.7.4.6; STER0929), a key enzyme for generating dTTP, which was not found in *S. mutans*[*50*], *S. sanguinis*[*51*], *S. gordonii*, and *S. pyogenes*. Genes encoding salvage enzymes were identified for the utilization and recycling of pre-formed nucleobases and nucleosides for nucleotide synthesis as well as sources of energy, carbon, and nitrogen. Purine salvage enzymes include adenine phosphoribosyltransferase (STER1190), hypoxanthine phosphoribosyltransferase (STER0013), and purine nucleoside phosphorylases (STER1072 and STER1075) allow salvage of adenine, guanine, and xanthine nucleobases. Meanwhile, genes encoding for a xanthine phosphorybosyltransferase and a putative xanthine permease (ψSTER1311 and ψSTER1312) that are present as a potential operon appears to be inactivated by frameshifts. The pyrimidine salvage pathway involves uracil phosphoribosyltransferase (STER0394) that converts uracil to UMP, which is complemented by two putative uracil uptake permeases (STER0375 and STER0556). The pyrimidine-nucleoside phosphorylase (EC 2.4.2.2) gene responsible for salvaging cytosine is truncated in all three *S. thermophilus* strains, whereas the gene is intact in the closely related *S. salivarius* and *S. mutans*. Nonetheless, the presence of a uridine kinase (STER1238) and a thymidine kinase (STER0792) potentially permits uridine, cytidine, and thymidine nucleosides as additional substrates for nucleotide synthesis.

While the biosynthetic pathways for the cofactors biotin, pantothenate, pyridoxine, thiamine, and riboflavin were incomplete, it is predicted that *S. thermophilus* is capable of synthesizing coenyzme A (CoA) and acyl carrier protein with L-cysteine or pre-formed pantothenate as precursors. As previously noted by van de Guchte *et al*. [52], the organism also possesses the complete pathway for folate biosynthesis from GTP or *p-*aminobenzoic acid (PABA) precursor, as well as *de novo* synthesis of PABA from chorismate (STER1554, STER0809).

### Protein secretion systems

The transport of proteins across the cytoplasmic membrane is a function of the general secretion (Sec) pathway. A second pathway determined as the TAT (twin arginine translocation) pathway secretes folded proteins. LMD-9 encodes components of both pathways that have been described in both LMG 18311 and CNRZ1066 [16]. As with the YidC orthologs in LMG 18311 and CNRZ1066, these proteins may interact with SecYEG, the membrane-spanning translocase, thereby potentially playing a role in membrane protein folding and assisting SecYEG to insert membrane proteins into the membrane [53].

The TAT translocase consists of either two, TatA and TatC, or three TatA, TatB and TatC integrated membrane components with the required energy provided by the proton motive force [54]. The TatP prediction tool was used to search for proteins encoded by LMD-9 that have a TAT signal peptide and TAT motif [30]. Seventeen proteins were identified, but only STER1023 has both a TAT signal peptide and TAT motif (Additional file 7), whereas the remaining 16 contained a potential TAT signal peptide only. STER1023 encodes for a probable iron-dependent peroxidase that may play a role in tolerance to oxidative stress and shows high identity (97%) to the corresponding ortholog in *S. salivarius*. BLAST analysis with the non-redundant database revealed orthologs in *S. salivarius* SK126 with high identity (~94%) for TatA and TatC. Orthologs for both TatA and TatC were found in the oral commensal and also opportunistic pathogenic streptococci *S. gordonii*, *Streptococcus mitis*, *S. sanguinis* and *Streptococcus parasanguinis* indicating that the TatAC rather than TatABC system is more common in streptococci.

### EPS gene clusters

**Figure 4:**
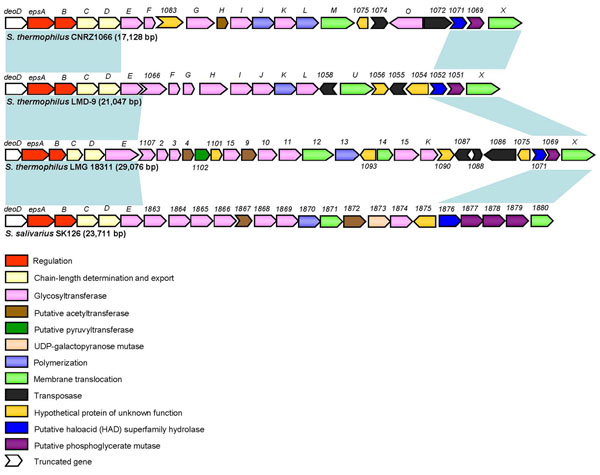
****The exopolysaccharide (EPS) biosynthetic gene clusters in *S. thermophilus* and *S. salivarius* SK126.**** ORFs were color-coded according to their prediction functions (see legend at lower left panel). Regions exhibiting sequence similarities among the *eps* clusters are shown in light blue-shaded boxes. The size of each *eps* cluster is indicated in bracket.

Similar to *S. thermophilus* CNRZ1066 and LMG 18311, LMD-9 has two EPS clusters, an *eps* cluster and a *rgp* cluster, the latter of which resembled the *rgp* cluster of *S. mutans* that encodes for the biosynthesis of rhamnose-glucose polysaccharide [16]. As in the case for most *S. thermophilus eps* operons [55], the *eps* clusters in all three *S. thermophilus* and *S. salivarius* SK126 showed conserved sequence (≥90% identity) and synteny confined to the terminal 5’ and 3’ regions of the clusters (Figure 4). All four *eps* clusters are flanked by *deoD* (encoding a purine nucleoside phosphorylase) and *bglH* gene (encoding a beta-glucosidase), noting that *bglH* is interrupted by frameshifts in all three *S. thermophilus* strains.

There is considerable variation in the sequence and little synteny in the central regions of the *eps* clusters in the *S. thermophilus* strains. Interestingly, the EPS biosynthetic components within this region of LMD-9 exhibited highest sequence similarity to the corresponding proteins in dairy associated *L. lactis* as well as microorganisms of human intestinal origin, such as *E. rectale*, *R. obeum*, and *L. gasseri* (R11, Additional file 3). The presence of two divergently-orientated transposase remnants together with the atypically low G + C content indicates that this region is likely acquired via LGT. Approximately 10 kb larger, the LMG 18311 *eps* cluster has a more complex mosaic structure, with several of the genes conserved with the *eps* genes from *S. thermophilus* IP6757. Meanwhile, CNRZ1066 has the most conservative *eps* cluster that is almost identical to the ones previously described in *S. thermophilus* MTC360, MR-2C and Sfi6 [56]

Although the physiological function of EPS production in *S. thermophilus* remains elusive, the marked variability of the *eps* regions and prominent presence of mobile elements at the vicinity of the *eps* clusters suggests the presence of unknown factors that drives the evolution and diversification of EPS among *S. thermophilus* strains via lateral genetic exchange. Aside from the main contribution of EPS to the texture and rheology of fermented products, EPS has also recently been associated with host immunostimulative effects [57]. It is likely that the diversity of the *eps* clusters may impart distinct immunomodulatory characteristics of the EPS produced by *S. thermophilus*.

### Specialized adaptation to the dairy environment. (i) Carbohydrate utilization

Lactose represents the principal carbohydrate component in milk. Like the majority of the *S. thermophilus* strains [58], LMD-9 was only able to ferment lactose, sucrose, and glucose, in order of preference, as assessed with API test and growth experiments (data not shown). Major gene decay among carbohydrate utilization genes occurred in LMD-9, a similar observation that was previously reported for strains LMG 18311 and CNRZ1066 [9]. In fact, the sets of intact carbohydrate utilization genes and pseudogenes are almost identical to those of LMG 18311 and CNRZ1066 (Table 2), indicating that gene degeneration occurred in the ancestor strain. Consequently, the limited repertoire of carbohydrate metabolic genes include three intact sugar hydrolases: a beta-galactosidase, a sucrose-6-phosphate hydrolase, and an intracellular alpha-amylase, in addition to (i) three intact PTS with predicted specificities for mannose/fructose, cellobiose, and sucrose; (ii) three putative sugar ABC transporters of unknown substrates, and (iii) two sugar permeases predicted for the uptake of glucose and lactose, respectively. The glucose/ribose permease (STER0891) gene in all three *S. thermophilus* strains is absent in *S. salivarius* SK126 and is located within a previously identified 17-kb LGT hot spot [9]. This sugar permease showed high identity (88 to 91%) to the orthologs in *L. rhamnosus*, *L. lactis* and *L. helveticus* that shared a close ecological niche. Meanwhile, all three *S. thermophilus* possessed complete pathways for the conversion of *N*-acetylglucosamine to substrates of glycolysis, amino acid metabolism and peptidoglycan biosynthesis. Bovine milk contains oligosaccharides, glycoproteins, and glycolipids that potentially serve as a source for *N-*acetylglucosamine. This suggests that aside from lactose, *S. thermophilus* is also equipped to utilize *N-*acetylglucosamine as an energy source during growth in milk.

**Table 2:** Carbohydrate transport systems and sugar hydrolases in *S. thermophilus* LMD-9

Locus Tag	Encoded protein	Homolog in LMG 18311	Homolog in CNRZ1066
**Phosphotransferase system (PTS)**			
STER0370	mannose/fructose PTS IID	stu0331	str0331
STER0371	mannose/fructose PTS IIC	stu0332	str0332
STER0372	mannose/fructose PTS IIAB	stu0333	str0333
STER1323	Cellobiose PTS IIC	stu1368	str1368
STER1710	Sucrose PTS EIIBCA	stu1734	str1734
ψSTER0237	glucose/*N*-acetylglucosamine PTS EIICBA, trunc.	stu0189	str0189
ψSTER0446	Fructose PTS IIA, trunc.	stu0401	str0401
ψSTER0544	Beta-glucoside PTS EIIABC, trunc.	stu0512	str0512
ψSTER1862	Trehalose PTS EIIABC, trunc.	stu1890	str1890
**ABC transporter**			
STER0856	ABC transporter, substrate-binding protein	stu0808	str0808
STER0857	Sugar ABC transporter, ATPase component	stu0809	str0809
STER0858	Sugar ABC transporter, permease component	stu0810	str0810
STER0859	Sugar ABC transporter, permease component	stu0811	str0811
STER1153	ABC transporter, ATPase component	stu1188	str1188
STER1154	Ribose/xylose/arabinose ABC transporter, permease component	stu1189	str1189
STER1155	ABC transporter, substrate-binding protein	stu1190	str1190
ψSTER0249	Ribose/xylose/arabinose ABC transporter, permease component, trunc.	stu0201	str0201
**Sugar permease**			
STER0891	Glucose/ribose permease	stu0855	str0855
STER1367	Lactose permease, LacS	stu1398	str1398
**Sugar hydrolase**			
STER1366	Beta-galactosidase, LacZ	stu1397	str1397
STER1500	Alpha-amylase (cytoplasmic)	stu1542	str1542
STER1711	Sucrose-6-phosphate hydrolase	stu1735	str1735
ψSTER1029	Putative pullulanase, trunc.		
ψSTER1049	Beta-glucosidase, trunc.	stu1064	str1064
ψSTER1594	6-phospho-beta-glucosidase, trunc.	stu1631	str1631
ψSTER1861	Trehalose 6-phosphate hydrolase, trunc.	stu1888	str1888

The *gal-lac* gene clusters are conserved in *S. thermophilus* and *S. salivarius*[*59*], and analogous *lac-gal* gene clusters are also present in the sequenced strains of *L. lactis*, *Lactobacillus acidophilus*, *Lactobacillus johnsonii*, *Lactobacillus plantarum* and *Lactobacillus salivarius*. However, *L. bulgaricus*, a companion culture for *S. thermophilus* during yogurt fermentation, only has a *lacSZ* operon. While absent in *S. pyogenes* and *Streptococcus uberis*, the *galRKTE* gene cluster is relatively conserved in the other sequenced streptococcal species. The *galM* gene encoding an aldose 1-epimerase is found only in strains of *S. thermophilus*, *S. salivarius* and *S. equi*. The GalM orthologs from *S. equi* strains, however, showed only moderate sequence homology (50% sequence identity) with the corresponding proteins from the former two species.

Orthologs of the *S. thermophilus* cytoplasmic alpha-amylase were found in most sequenced streptococci (with 60-70% sequence identity) with the exception of *S. pyogenes* and *S. suis* strains. Due to the inability of LMD-9 to ferment starch, the intracellular alpha-amylase is less likely to have a direct role in starch degradation, as previously described for *Streptococcus bovis* and *S. mutans*. The intracellular amylases of *S. bovis* strains were shown to be essential for rapid cell growth [60], or acted as maltotriose-producing endo-amylase [61]. In *S. mutans*, this enzyme is involved in the accumulation of glycogen-like intracellular polysaccharide during growth on maltose [62]. Meanwhile, remnants of a putative pullulanase, a trehalose 6-phosphate hydrolase, and two beta-glucosidase-encoding genes are also present in the genome, the latter of which likely explained the inability of LMD-9 to utilize cellobiose despite the presence of an intact orphan cellobiose PTS EIIC-encoding gene. Overall, the majority of the pseudogenes are involved in the transport and hydrolysis of glucosides, suggesting a possible niche transit of *S. thermophilus* ancestor strain from plant to dairy environment.

### (ii) Proteolytic system

Milk is poor in free amino acids as its rich protein content is present mainly in the form of casein. Although LMD-9 is predicted to be capable of synthesizing most of the amino acids, its genome encodes an intact cell wall-associated proteinase PrtS, an abundance of cytoplasmic peptidases and transport systems that allows the utilization of exogeneous protein source in milk. As previously mentioned, the PrtS is encoded in one of the LMD-9 unique regions (R9; Additional file 3) and is highly conserved (95% identity) with the PrtS of *S. suis*. Although recent work suggested that PrtS contributes to the virulence of *S. suis*[*63*], it is well established that in *S. thermophilus* strains, the primary role of PrtS involved the cleavage of casein to oligopeptides, a clear function related to its dairy adaptation. Previous study with the PrtS of *S. thermophilus* CNRZ385 showed that the proteinase is essential for growth in milk unless co-cultured with *L. bulgaricus*[64].

The LMD-9 genome possessed all 12 cytoplasmic peptidases (*pepA*, *pepC*, *pepF*, *pepM*, *pepN*, *pepO*, *pepP*, *pepQ*, *pepS*, *pepT*, *pepV*, *pepX*) present in both LMG 18311 and CNRZ1066 [16] strains that potentially provide flexibility in peptide substrate specificities. Unlike most lactic acid bacteria where some peptidase genes formed operon structures with adjacent amino acid/peptide transporter genes, none of the peptidase-encoding genes in *S. thermophilus* is located at the vicinity of any amino acid/peptide uptake system. Nine complete ABC transport systems are complemented by five permease porters for the uptake of amino acids, oligopeptides and polyamines, including: two oligopeptide *opp* ABC transporters (STER0142-0145, STER1405-1409), three polar amino acids ABC transporters (STER0905-0907, STER1539-1542, STER1617-1619), one ABC transporter (STER0398-0402) and two symporters for branched-chain amino acids (STER0993, STER1315), one glutamine ABC transporter (STER1461-1462), and one spermidine/putrescine ABC transporter (STER1493-1496). As mentioned previously, one of the *opp* transport systems (STER0142-0145) is located within a 10.2-kb unique region (R2; Additional file 3) of LMD-9 that shared moderate homology with distant species of *Bifidobacterium*. This finding has led us to speculate that LGT events potentially occurred beyond the milk niche.

### (iii) Stress response and transcriptional regulation

*S. thermophilus* often encounter physiological stresses caused by adverse conditions during commercial production of yogurt and cheeses as well as preservation of starter cultures. These include low pH, changes in osmolarity and temperatures, oxidative stress, nutrient limitation and competition in mixed culture environments. The ability of strains to adapt and perform in these industrial stress conditions is crucial to the consistency and organoleptic properties of the final products. The LMD-9 genome encodes a proton translocating F_0_F_1_-ATPase system (STER0515-0522) and a putative cation transport ATPase (STER1107) that likely contribute to internal pH homeostasis in high acidity environments. To cope with oxidative stress, LMD-9 possesses a Mn-superoxide dismutase (STER0762) for the elimination of reactive oxygen species along with disulfide-reducing pathway enzymes, including a glutathione reductase (STER0447), thioredoxins (STER1776, STER1825) and thioredoxin reductases (STER1382, STER1615). Other putative proteins involved in oxidative stress tolerance include an NADH oxidase (STER1258) that potentially plays a role in the removal of oxygen and two methionine sulfoxide reductases (STER1314 and STER1596) that serve housekeeping protein function via methionine sulfoxide reduction in proteins.

The classical class I heat-shock genes encoding the conserved chaperone complexes GrpE-DnaK-DnaJ and GroES-GroEL were identified, with the HrcA repressor-encoding gene located upstream of the *grpE-dnaK-dnaJ* operon. Other heat-shock genes include the class III CtsR repressor (STER0108), along with four Clp-ATPase chaperones (STER0109, STER0625, STER0648, STER1578) and a ClpP proteolytic component (STER0395) that degrade nonfunctional proteins. The pSTER_B plasmid of LMD-9, a pC194 family rolling circle replicating plasmid, encodes a 143-amino acid small heat shock protein (sHsp; pSTER_B1) that is conserved (≥ 81% identity) in several other plasmid-bearing *S. thermophilus* strains [65,66]. In fact, pSTER_B1 is identical to the sHsp from pER1-1 of *S. thermophilus* ER1 along with the upstream conserved CtsR regulatory region [65], and the RepA deduced proteins from both plasmids shared 99% sequence identity. Expression of these plasmid-encoded sHsp has been shown to be upregulated at low pH or elevated temperatures, suggesting that pSTER_B potentially provides multitude cross-protections that enhance the survival of LMD-9 under various stress conditions. Meanwhile, *S. thermophilus* has two cold-shock protein genes (STER0879 and STER0880) arranged in tandem and both deduced proteins shared only 50% identity. No homolog of STER0879 was found among other streptococci, whereas STER0880 is conserved mainly among the pyogenic group of streptococci (~ 80% identity). Interestingly, STER0879 and STER0880 showed highest similarity to the tandem *cspCD* genes in *L. lactis* (95% identity), which was induced by cold shock at 10°C [67]. While one glycine-betaine transporter, OpuD (STRSA0001_1242) was found in the *S. salivarius* SK126 draft genome, no specific transporter or biosynthetic pathway for compatible solutes was detected in the *S. thermophilus* genomes. Nevertheless, several Trk-type K^+^ transporter homologs are present (STER0311-STER0312 and STER0350-STER0351) that may play a role in cytoplasmic osmoregulation.

The ability of microorganisms to survive and adapt to changing environments relies on the intricate sensing and signal transduction mechanisms of two-component regulatory systems. Hols *et al.*[*16*] previously noted that all three *S. thermophilus* sequenced strains have the same set of 20 two-component regulatory system (2CRS) genes. Closer examination of the LMD-9 2CRS gene sets revealed six sets of 2CRS consisting of both histidine protein kinase (HK) and response-regulator (RR) genes adjacent to each other (Additional file 8), compared to seven and eight sets of 2CRS that were identified in CNRZ1066 and LMG 18311 strains, respectively. In comparison, the *S. salivarius* SK126 draft genome has 12 sets of HK-RR systems, reflecting its adaptation to a more complex lifestyle.

### (iv) Cell surface proteins

Cell surface proteins can be anchored to the cell wall peptidoglycan through the action of sortase which recognizes an LPXTG binding motif preceding a hydrophobic region at the C-terminus of the precursor proteins. The LMD-9 strain encodes for an intact sortase, SrtA (STER1255) which presents as a pseudogene in both LMG 18311 and CNRZ1066 [16]. In addition, three proteins with cell sorting motifs are pseudogenes in LMG 18311 and CNRZ1066, whereas LMD-9 encodes for three sortase-dependant proteins in addition to two pseudogenes containing the sortase recognition motif. The three intact proteins which all contain an LPNTG motif are the aforementioned PrtS and Mub, in addition to a cyclo-nucleotide phosphodiesterase CpdB (STER0198). Inactivation of the *srtA* gene in *S. sanguinis*[68] and *S. uberis*[69] demonstrated its role in bacterial colonization. The sortase from LMD-9 showed high identity (~90%) with the sortase from *S. salivarius* SK126. In addition to a second sortase, SrtB, *S. salivarius* also encodes for 420 proteins with signal peptide sequences compared to 271 in LMD-9 (Additional file 7), reflecting the roles of secreted proteins in different environmental niches of these closely related commensal versus dairy domesticated species*.*

Unlike PrtS and CpdB, the putative Mub does not have a SPaseI signal sequence at its N-terminal end, but rather the YSIRK Gram-positive signal peptide [42] ([YF]SIRKxxxGxxS[VIA]; IPR005877), in this case YGIRKFSFGAASVAIA. There is little sequence homology to MucBP domain proteins from *S. salivarius*, however Mub shares 66% identity with a conserved hypothetical protein from *L. fermentum* that also contains a single MucBP binding domain and an LPXTG motif. The presence of this gene in LMD-9 may represent an ancestral remnant as Mubs are not encoded in the other *S. thermophilus* genomes. The loss of these functions in dairy bacteria has also been demonstrated in other lactic acid bacteria that have evolved to a specific ecological niche. For example, the small intestinal-associated *L. acidophilus* encodes for several Mubs compared to none in the closely related cheese starter strain of *L. helveticus*, demonstrating reductive evolution in their dairy environment [70]. Mubs in the case of streptococci may be important for pathogenesis of these strains, providing an advantage in regard to colonization. Loss of these virulence-like features is a common trait observed in *S. thermophilus* strains [9].

The third cell surface protein, CpdB, is believed to convert non-transportable nucleotides produced by RNaseI to nucleosides which can then readily enter the cell and be utilized as a carbon source. CpdB has recently been reported as a virulence factor and was upregulated under iron starvation in *S. suis*[71]. Identification of this orthologous gene (SntA) in *S. suis* revealed a tripeptide RGD motif in CpdB [72]. This RGD motif has been demonstrated to bind to integrins of mammalian cells and hence may play a role in host interactions. Interestingly the CpdB of LMD-9 also contains an RGD motif starting at amino acid position 519. Both LMG 18311 and CNRZ1066 possessed a truncated CpdB that has an LPXTG motif but lacks an N-terminal signal peptide [16]. The CpdB of LMD-9 shows high homology (91%) to the CpdB of *S. salivarius* and intermediate identity to orthologs from *S. sanguinis* (60%) and *S. suis* (56%)*.* Compared to LMG 18311 and CNRZ1066, it is tempting to suggest that LMD-9 has as yet not undergone as extensive genomic decay in terms of genes that may play a beneficial role in the gastrointestinal tract such as mucin binding and utilization of metabolites, which has also been observed for probiotic lactobacilli [70]. This is in addition to an intact sortase in LMD-9 that allows processing of PrtS for cell surface exposure that potentially confer an advantage for growth of LMD-9 in milk.

### (v) Response to bacteriophage infection

Several mechanisms exist within the *S. thermophilus* genome to combat bacteriophage infections. Bacteriophage resistance is usually conferred via non-specific point mutations in genes involving cell receptor sites [73]. In addition to other, more traditional bacteriophage resistance mechanisms, *S. thermophilus* LMD-9 possesses three separate CRISPR loci, and 14 *cas* genes [18]. The activity of CRISPR genes at the onset of phage DT1 exposure was determined by DNA microarray analysis for each of the 14 known CRISPR genes, spread among 3 separate loci in the *S. thermophilus* LMD-9 genome (Figure 1, circle 10). The phage response arrays indicated a transient increase in global gene expression, including that of CRISPR genes, 5 minutes post infection, followed by a decrease in gene expression (Additional file 9).

The increased expression of the CRISPR1 locus (specifically, *cas2* and *cas1* genes) is indicative of their higher activity during the phage response. CRISPR1 is known to have repeat degeneracy within the CRISPR gene sequence, with spacer size more highly conserved and in the highest number when compared to the other two loci [18]. This increase in spacer number and conservation in CRISPR1 is likely evidence of an effective mechanism to integrate novel spacers when faced with novel phage infection [74]. CRISPR3 plays a lesser role in phage response, and likewise less gene expression was observed. CRISPR2 has not been associated with active phage response [18,74].

### (vi) Protocooperation with *Lactobacillus bulgaricus*

During yogurt fermentation, protocooperation between *S. thermophilus* and *L. bulgaricus* results in rapid acidification of milk. As *S. thermophilus* is used in conjunction with *L. bulgaricus* for yogurt fermentations, the sequencing of both genomes revealed additional symbiotic or “protocooperative” relationship between both strains. The advantages conferred between both species have been discussed previously with the release of the *L. bulgaricus* genome sequence [52]. These include an extracellular cell wall bound proteinase (PrtB) encoded by *L. bulgaricus* and the biosynthetic pathways of folate, *p*-aminobenzoic acid and ornithine by *S. thermophilus*[52]. More recently, *in silico* analysis of both genomes predicted genes acquired by LGT for both species [38]. These LGT events included the transfer of an EPS biosynthesis cassette from *S. thermophilus* to *L. bulgaricus*, and genes for the metabolism of sulfur-containing amino acids from *L. bulgaricus* (or *L. helveticus*) to *S. thermophilus*.

*	In silico* analysis of LMD-9 revealed the presence of a gene (STER0938) encoding a putative enzyme with conserved domain (COG0010) and a pfam domain (00491) related to the arginase family of enzymes. The putative arginase/agmatinase is likely involved in the biosynthesis of polyamine putrescine by the conversion of L-arginine to L-ornithine and urea, and thus may contribute to protocooperation with *L. bulgaricus* through polyamine metabolism. This gene is uniquely present in *S. thermophilus* (Additional file 4) as no ortholog is found in other streptococci, *L. lactis* or lactobacilli. In fact, the next best sequence match in the current database was less than 30% identity with orthologs from an archaea species isolated from metagenomic analysis of soil. Its low G + C composition (28.2%) confirms that the gene was horizontally acquired, presumably from another low GC microorganism.

	The transcriptomics of *S. thermophilus* LMD-9 during milk fermentation in co-culture with *L. bulgaricus* revealed significant regulation of several amino acid metabolism and transport genes compared to the control (data not shown). This result was expected, as the presence of *L. bulgaricus* and its extracellular protease would increase the level of hydrolyzed proteins that would be available for use once the free amino acids from the milk media were depleted. Herve-Jimenez *et al*. [75] likewise showed that there was an upregulation of amino acid biosynthesis and nucleic acid metabolism in *S. thermophilus* when grown in co-culture with *L. bulgaricus*.

## Conclusions

Although milk is a rich growth medium for many microorganisms, bacteria that grow and compete well in the milk environment must, at minimum, be able to use lactose as an energy source and milk protein as a source of amino acids. The adaptation of *S. thermophilus* LMD-9 to the milk environment is reflected by several observations from genomic and transcriptome analyses, including specialized systems for metabolizing lactose, the general absence of other carbohydrate metabolic systems, the presence of amino acid and peptide scavenging machinery, and numerous stress response and host defense mechanisms. Functional genome distribution analysis showed a close functional relationship between *S. thermophilus* and *S. salivarius*. Although the genome of *S. thermophilus* reflects a more simplified lifestyle compared to *S. salivarius*, both species shared several unique gene features (e.g. urease biogenesis, lactose metabolism) that are not universally present in other streptococci, which were possibly acquired by clonal populations from their natural ecosystems. Meanwhile, almost half of *S. thermophilus* LMD-9 unique genes have no orthologous counterpart among other streptococci. This indicates that LGT, the majority of which were likely mediated via transposable elements, served an important role in shaping the unique gene repertoire that potentially contribute to the fitness of LMD-9 in its specialized niches. The degree of sequence similarity of some of these alien genes, e.g. those involved in EPS biosynthesis and peptide transporters with microorganisms naturally found in the human intestinal environment suggests multiple transient habitats of LMD-9 beyond the restricted dairy environment. Compared to LMG 18311 and CNRZ1066, the notable presence of intact cell surface factors (mucus-binding protein, cyclo-nucleotide phosphodiesterase) in LMD-9 suggests that the latter strain has as yet not undergone as extensive genomic decay with regards to genes that may contribute to the interaction with human intestinal epithelium. This is in addition to unique EPS signature and functional bacteriocin production machinery that potentially expand the probiotic attributes of LMD-9.

## Competing interests

The authors declare that they have no competing interests.

## Authors’ contributions

YJG performed genome annotation, genome alignments, *in silico* and comparative genome analysis, and carbohydrate growth experiments. CG carried out microarray gene expression studies and transcriptome analysis. SOF performed *in silico* secretome and protocooperation analysis. EA conducted functional genome distribution analysis and comparative genome analysis. RH conceived the study, and participated in its design and coordination. All authors wrote, read and approved the final manuscript.

## Supplementary Material

Additional file 1 Sequenced *Streptococcus* strains included in the Functional Genome Distribution (FGD) analysis Click here for file

Additional file 2 Pseudogenes in the *S. thermophilus* LMD-9 genome Click here for file

Additional file 3 *S. thermophilus* LMD-9 specific gene regions that are absent in both CNRZ1066 and LMG 18311 strains Click here for file

Additional file 4 *S. thermophilus*-specific genes not present in *S. salivarius* SK126 draft genome Click here for file

Additional file 5 Unique genes among the sub-clusters of *S. pneumoniae* strains Click here for file

Additional file 6 Unique genes among the sub-clusters of *S. pyogenes* strains Click here for file

Additional file 7 ORFs with predicted signal peptide sequence in *S. thermophilus* LMD-9 Click here for file

Additional file 8 Two-component regulatory systems (2CRS) in *S. thermophilus* LMD-9 Click here for file

Additional file 9 CRISPR gene activity during phage infection Click here for file
