# Supplementary material for: Specialized adaptation of a lactic acid bacterium to the milk environment: the comparative genomics of Streptococcus thermophilus LMD-9
Source: Microb Cell Fact. 2011 Aug 30;10(Suppl 1):S22. doi: 10.1186/1475-2859-10-S1-S22 (PMC3231929; doi:10.1186/1475-2859-10-S1-S22)
Supplement: Additional file 1 — Sequenced Streptococcus strains included in the Functional Genome Distribution (FGD) analysis [file 1475-2859-10-S1-S22-S1.doc]

Additional file 1. Sequenced *Streptococcus* strains included in the Functional Genome Distribution (FGD) analysis

| *Streptococcus* species | Strain | Genome size (Mb) | G + C content | GenBank accession number |
| --- | --- | --- | --- | --- |
| *S. agalactiae* | NEM316 | 2.2 | 35.6 | [AL732656](http://www.ncbi.nlm.nih.gov/nuccore/AL732656) |
|  | 2603V/R | 2.2 | 35.7 | [AE009948](http://www.ncbi.nlm.nih.gov/nuccore/AE009948) |
|  | A909 | 2.1 | 35.6 | [CP000114](http://www.ncbi.nlm.nih.gov/nuccore/CP000114) |
|  |  |  |  |  |
| *S. equi* subsp. *equi* | 4047 | 2.3 | 41.3 | [FM204883](http://www.ncbi.nlm.nih.gov/nuccore/FM204883) |
| *S. equi* subsp. *zooepidemicus* | H70 | 2.1 | 41.5 | [FM204884](http://www.ncbi.nlm.nih.gov/nuccore/FM204884) |
|  | MGCS10565 | 2.0 | 41.8 | [CP001129](http://www.ncbi.nlm.nih.gov/nuccore/CP001129.1) |
|  |  |  |  |  |
| *S. gordonii* | [Challis substr. CH1](http://www.ncbi.nlm.nih.gov/genomeprj/66) | 2.2 | 40.5 | [CP000725.1](http://www.ncbi.nlm.nih.gov/nuccore/CP000725.1) |
|  |  |  |  |  |
| *S. mutans* | UA159 | 2.0 | 36.8 | [AE014133](http://www.ncbi.nlm.nih.gov/nuccore/AE014133.1) |
|  |  |  |  |  |
| *S. pneumoniae* | R6 | 2.0 | 39.7 | [AE007317](http://www.ncbi.nlm.nih.gov/nuccore/AE007317.1) |
|  | D39 | 2.0 | 39.7 | [CP000410](http://www.ncbi.nlm.nih.gov/nuccore/CP000410.1) |
|  | CGSP14 | 2.2 | 39.5 | [CP001033](http://www.ncbi.nlm.nih.gov/nuccore/CP001033.1) |
|  | TIGR4 | 2.2 | 39.7 | [AE005672](http://www.ncbi.nlm.nih.gov/nuccore/AE005672.3) |
|  | 70585 | 2.2 | 39.7 | [CP000918](http://www.ncbi.nlm.nih.gov/nuccore/CP000918.1) |
|  | [Hungary19A-6](http://www.ncbi.nlm.nih.gov/genomeprj/28035) | 2.2 | 39.6 | [CP000936](http://www.ncbi.nlm.nih.gov/nuccore/CP000936.1) |
|  | [ATCC 700669](http://www.ncbi.nlm.nih.gov/genomeprj/31233) | 2.2 | 39.5 | [FM211187](http://www.ncbi.nlm.nih.gov/nuccore/FM211187.1) |
|  | JJA | 2.1 | 39.7 | [CP000919](http://www.ncbi.nlm.nih.gov/nuccore/CP000919.1) |
|  | G54 | 2.1 | 39.7 | [CP001015](http://www.ncbi.nlm.nih.gov/nuccore/CP001015.1) |
|  | P1031 | 2.1 | 39.7 | [CP000920](http://www.ncbi.nlm.nih.gov/nuccore/CP000920.1) |
|  | [Taiwan19F-14](http://www.ncbi.nlm.nih.gov/genomeprj/28037) | 2.1 | 39.8 | [CP000921](http://www.ncbi.nlm.nih.gov/nuccore/CP000921.1) |
|  |  |  |  |  |
| *S. pyogenes* | MGAS10270 | 1.9 | 38.4 | [CP000260](http://www.ncbi.nlm.nih.gov/nuccore/CP000260.1) |
|  | MGAS6180 | 1.9 | 38.4 | [CP000056](http://www.ncbi.nlm.nih.gov/nuccore/CP000056.1) |
|  | MGAS10750 | 1.9 | 38.3 | [CP000262](http://www.ncbi.nlm.nih.gov/nuccore/CP000262.1) |
|  | MGAS5005 | 1.8 | 38.5 | [CP000017](http://www.ncbi.nlm.nih.gov/nuccore/CP000017.1) |
|  | MGAS2096 | 1.9 | 38.7 | [CP000261](http://www.ncbi.nlm.nih.gov/nuccore/CP000261.1) |
|  | MGAS9429 | 1.8 | 38.5 | [CP000259](http://www.ncbi.nlm.nih.gov/nuccore/CP000259.1) |
|  | MGAS315 | 1.9 | 38.6 | [AE014074](http://www.ncbi.nlm.nih.gov/nuccore/AE014074.1) |
|  | SSI-1 | 1.9 | 38.6 | [BA000034](http://www.ncbi.nlm.nih.gov/nuccore/BA000034.2) |
|  | MGAS10394 | 1.9 | 38.7 | [CP000003](http://www.ncbi.nlm.nih.gov/nuccore/CP000003.1) |
|  | MGAS8232 | 1.9 | 38.5 | [AE009949](http://www.ncbi.nlm.nih.gov/nuccore/AE009949.1) |
|  | Manfredo | 1.8 | 38.6 | [AM295007](http://www.ncbi.nlm.nih.gov/nuccore/AM295007.1) |
|  | M1 GAS (SF370) | 1.9 | 38.5 | [AE004092](http://www.ncbi.nlm.nih.gov/nuccore/AE004092.1) |
| *S. salivarius* | SK126 | (Draft) | 40.1 | [ACLO00000000](http://www.ncbi.nlm.nih.gov/nuccore/ACLO00000000) |
|  |  |  |  |  |
| *S. sanguinis* | SK36 | 2.4 | 43.4 | [CP000387](http://www.ncbi.nlm.nih.gov/nuccore/CP000387.1) |
|  |  |  |  |  |
| *S. suis* | 05ZYH33 | 2.1 | 41.1 | [CP000407](http://www.ncbi.nlm.nih.gov/nuccore/CP000407.1) |
|  | 98HAH33 | 2.1 | 41.1 | [CP000408](http://www.ncbi.nlm.nih.gov/nuccore/CP000408.1) |
|  |  |  |  |  |
| *S. thermophilus* | LMD-9 | 1.9 | 39.1 | [CP000419](http://www.ncbi.nlm.nih.gov/nuccore/CP000419.1) |
|  | CNRZ1066 | 1.8 | 39.1 | [CP000024](http://www.ncbi.nlm.nih.gov/nuccore/CP000024.1) |
|  | LMG 18311 | 1.8 | 39.1 | [CP000023](http://www.ncbi.nlm.nih.gov/nuccore/CP000023.1) |
|  |  |  |  |  |
| *S. uberis* | 0140J | 1.9 | 36.6 | [AM946015](http://www.ncbi.nlm.nih.gov/nuccore/AM946015.1) |
