# Supplementary material for: Specialized adaptation of a lactic acid bacterium to the milk environment: the comparative genomics of Streptococcus thermophilus LMD-9
Source: Microb Cell Fact. 2011 Aug 30;10(Suppl 1):S22. doi: 10.1186/1475-2859-10-S1-S22 (PMC3231929; doi:10.1186/1475-2859-10-S1-S22)
Supplement: Additional file 2 — Pseudogenes in the S. thermophilus LMD-9 genome [file 1475-2859-10-S1-S22-S2.doc]

Additional file 2. Pseudogenes in the *S. thermophilus* LMD-9 genome

| COG | Locus tag | Gene description |  |
| --- | --- | --- | --- |
| **[C] Energy production and conversion** | | |  |
|  | STER1859 | Alcohol-acetaldehyde dehydrogenase, trunc. | FS^a^ |
| **[E] Amino acid transport and metabolism** | | | |
|  | STER0223 | Oligopeptide ABC transporter substrate-binding protein, trunc. | FS |
| (EP) | STER0224 | Dipeptide/oligopeptide ABC transporter permease protein, trunc. | FS |
| (EP) | STER0225 | Dipeptide/oligopeptide ABC transporter ATPase, trunc. | FS |
|  | STER0341 | Succinyl-diaminopimelic descuccinlyasadipeptidase, trunc. | FS |
|  | STER0595 | Alanine dehydrogenase, trunc. | FS |
|  | STER0707 | Hypothetical protein, trunc. | FRAGM |
|  | STER0843 | Spermidine/putrescine ABC transporter permease component, trunc. | FRAGM |
|  | STER0878 | Dipeptidase, trunc. | FS |
|  | STER0928 | Putative methionine synthase II (cobalamin-independent), trunc. | FS |
| (ET) | STER0985 | Amino acid ABC transporter permease, trunc. | FS |
|  | STER1045 | Putative platelet activating factor, trunc. | FS |
|  | STER1177 | Putative GDSL-like lipase/acylhydrolase, trunc. | FS |
|  | STER1414 | Oligopeptide ABC transporter ATPase, trunc. | FS |
|  | STER1795 | Oligopeptide ABC transporter substrate-binding protein, trunc. | FS |
|  | STER1952 | Methionine synthase II, trunc. | FS |
| **[F] Nucleotide transport and metabolism** | | |  |
|  | STER0853 | Thymidine phosphorylase, trunc. | FS |
|  | STER0854 | Deoxyribose-phosphate aldolase, trunc. | FS |
|  | STER0989 | Putative 5'-nucleosidase, trunc. | FS |
|  | STER0991 | Putative AraC family transcriptional regulator, trunc. | FS |
|  | STER1073 | Glutamine amidotransferase, trunc. | FS |
|  | STER1311 | Xanthine phosphoribosyltransferase, trunc. | FS |
|  | STER1312 | Xanthine/uracil permease, trunc. | FS |
|  | STER1964 | Putative NUDIX family pyrophosphatase, trunc. | FS |
| **[G] Carbohydrate transport and metabolism** | | |  |
|  | STER0146 | Putative bacteriocin ABC exporter accessory protein, trunc. | FRAGM |
|  | STER0237 | Glucose PTS EIICBA, trunc. | FS |
|  | STER0249 | ABC transporter substrate-binding protein, trunc. | FRAGM |
| (GT) | STER0446 | Fructose PTS EIIABC, trunc. | FS |
|  | STER0544 | Beta-glucoside PTS EIIABC components, trunc. | FS |
| (GEPR) | STER0671 | MFS multidrug resistance efflux pump, trunc. | FS |
|  | STER1029 | Putative pullulanase, trunc. | FS |
|  | STER1049 | Putative beta-glucosidase, trunc. | FS |
|  | STER1051 | Putative phosphoglycerate mutase, trunc. | FS |
|  | STER1211 | Probable exopolysaccharide biosynthesis protein, trunc. | FS |
|  | STER1316 | Mannose-6-phosphate isomerase, trunc. | FS |
|  | STER1594 | 6-phospho-beta-glucosidase, trunc. | FRAGM |
|  | STER1861 | Trehalose-6-phosphate hydrolase, trunc. | FS |
|  | STER1862 | Trehalose PTS EIIABC components, trunc. | FS |
| **[H] Coenzyme transport and metabolism** | | |  |
|  | STER1261 | Pyridoxamine kinase, trunc. | FS |
|  | STER1878 | Gamma-glutamylcysteine synthetase GshA, trunc. | FS |
| **[I] Lipid metabolism** | | |  |
| (IQR) | STER0931 | Acetoin reductase, trunc. | FS |
| (IR) | STER1956 | Diacylglycerol kinase catalytic domain protein, trunc. | FS |
| **[J] Translation, ribosomal structure and biogenesis** | | |  |
|  | STER0512 | GNAT family acetyltransferase, trunc. | FRAGM |
| **[K] Transcription** | | |  |
|  | STER0099 | MarR family transcriptional regulator, trunc. | FS |
|  | STER0129 | Putative transcriptional regulator, trunc. | FRAGM |
| (KG) | STER0267 | DeoR family transcriptional regulator, trunc. | FRAGM |
|  | STER0270 | Putative MarR family transcriptional regulator, trunc. | FS |
| (KR) | STER0287 | GNAT family acetyltransferase, trunc. | FRAGM |
|  | STER0657 | Putative transcriptional regulator, trunc. | FRAGM |
|  | STER0665 | BglG family transcriptional antiterminator, trunc. | FS |
|  | STER1018 | Putative maltose operon transcriptional repressor, LacI family, trunc. | FRAGM |
| (KG) | STER1490 | Transcriptional regulator, trunc. | FS |
|  | STER1562 | Putative transcriptional regulator, trunc. | FRAGM |
|  | STER1863 | Trehalose operon repressor, trunc. | FS |
|  | STER1922 | Putative transcriptional regulator, trunc. | FRAGM |
|  | STER1966 | Transcriptional regulator (PadR family), trunc. | FS |
| **[L] DNA replication, recombination, and repair** | | |  |
|  | STER0015 | Putative transposase, trunc. | FRAGM |
|  | STER0018 | Transposase, trunc. | FS |
|  | STER0044 | Transposase (IS1193), trunc. | FS |
|  | STER0056 | Transposase, trunc. | FS |
|  | STER0101 | Transposase, trunc. | FS |
|  | STER0132 | Transposase, trunc. | FS |
|  | STER0152 | Transposase, trunc. | FRAGM |
|  | STER0188 | IS1193 transposase, trunc. | FRAGM |
|  | STER0254 | Transposase, trunc. | FS |
|  | STER0255 | Transposase, trunc. | FS |
|  | STER0290 | Transposase, trunc. | FRAGM |
|  | STER0291 | Transposase, trunc. | FRAGM |
|  | STER0364 | Transposase, trunc. | FS |
|  | STER0366 | Transposase, trunc. | FS |
|  | STER0441 | IS1216 transposase, trunc. | FRAGM |
|  | STER0443 | IS1167 transposase, ISL3 family, trunc. | FRAGM |
|  | STER0569 | Transposase, trunc. | FS |
|  | STER0570 | Putative transposase, trunc. | FRAGM |
|  | STER0706 | Site-specific DNA-methyltransferase (adenine-specific) LlaI, trunc. | FRAGM |
|  | STER0763 | Transposase (IS1193), trunc. | FS |
|  | STER0785 | IS1193 transposase, trunc. | FRAGM |
|  | STER0806 | Putative transposase, trunc. | FS |
|  | STER0807 | Transposase, trunc. | FRAGM |
|  | STER0812 | Putative integrase, trunc. | FRAGM |
|  | STER0842 | Transposase, IS204/IS1001/IS1096/IS1165/IS1167 fam., trunc. | FRAGM |
|  | STER0867 | Putative IS1193 transposase, trunc. | FS |
|  | STER0900 | Putative transposase, trunc. | FS |
|  | STER0911 | Type III R/M system methylation subunit, trunc. | FRAGM |
|  | STER0942 | IS1193 transposase, trunc. | FS |
| (LR) | STER0973 | CRISPR-associated Csm1 family protein, trunc. | FS |
|  | STER1055 | Transposase, trunc. | FRAGM |
|  | STER1058 | Transposase, trunc. | FRAGM |
|  | STER1086 | Putative transposase, trunc. | FRAGM |
|  | STER1209 | Transposase, trunc. | FS |
|  | STER1210 | Transposase, trunc. | FS |
|  | STER1275 | Putative DNA alkylation repair protein, trunc. | FRAGM |
|  | STER1277 | Putative transposase IS1193, trunc. | FS |
|  | STER1373 | Transposase, trunc. | FRAGM |
|  | STER1380 | Transposase IS1193, trunc. | FRAGM |
|  | STER1415 | Putative Mg-dependent DNase, trunc. | FRAGM |
|  | STER1417 | Transposase, trunc. | FRAGM |
|  | STER1418 | Transposase, trunc. | FS |
|  | STER1486 | Methylated-dna--protein-cysteine methyltransferase, trunc. | FRAGM |
|  | STER1602 | Transposase, trunc. | FRAGM |
|  | STER1754 | Transposase, trunc. | FS |
|  | STER1756 | Transposase, trunc. | FRAGM |
|  | STER1757 | Transposase, trunc. | FS |
|  | STER1760 | Transposase, trunc. | FS |
|  | STER2009 | Putative IS1193 transposase, trunc. | FS |
|  | STER1786 | Putative transposase, trunc. | FRAGM |
|  | STER1871 | Putative phage integrase, trunc. | FRAGM |
|  | STER1875 | IS30 family transposase, trunc. | FRAGM |
|  | STER1917 | Transposase, trunc. | FS |
| **[M] Cell envelope biogenesis, outer membrane** | | |  |
|  | STER0110 | D-alanyl-D-alanine carboxypeptidase, trunc. | FS |
|  | STER0154 | Similar to capsule biosynthesis protein, trunc. | FS |
|  | STER0667 | Diacylglycerol kinase, trunc. | FRAGM |
|  | STER1043 | D-alanyl-D-alanine carboxypeptidase, trunc. | FS |
|  | STER1066 | Putative rhamnosyltransferase, trunc. | FRAGM |
|  | STER1327 | UDP-glucose 4-epimerase, trunc. | FRAGM |
|  | STER1450 | Probable large conductance mechanosensitive channel protein, trunc. | FRAGM |
|  | STER1593 | Glycosyltransferase, trunc. | FRAGM |
| **[N] Cell motility and secretion** | | |  |
| (NU) | STER0495 | Conserved hypothetical protein (containing GBS Bsp-like repeat), trunc. | FS |
| (NU) | STER0497 | Conserved hypothetical protein (containing GBS Bsp-like repeat), trunc. | FS |
| **[O] Posttranslational modification, protein turnover, chaperones** | | |  |
|  | STER1492 | SPFH domain/Band 7 family protein, trunc. | FS |
| (OC) | STER1638 | Probable immunity/modification protein, trunc. | FS |
| **[P] Inorganic ion transport and metabolism** | | |  |
|  | STER0389 | Putative cation transporter P-type ATPase, trunc. | FRAGM |
|  | STER0536 | Putative Na+/phosphate symporter, trunc. | FRAGM |
|  | STER0957 | Putative ferrichrome ABC transporter substrate-binding protein, trunc. | FRAGM |
|  | STER1074 | Chloride channel protein EriC, trunc. | FS |
|  | STER1226 | Putative zinc/iron permease, trunc. | FS |
|  | STER1391 | Putative potassium channel, trunc. | FRAGM |
|  | STER1807 | Putative formate/nitrite transporter, trunc. | FS |
| **[Q] Secondary metabolites biosynthesis, transport and catabolism** | | |  |
|  | STER271 | Acyl-CoA thioesterase I, trunc. | FS |
| **[R] General function prediction only** | | |  |
|  | STER0186 | Aminoacylase/N-acyl-L-amino acid amidohydrolase., trunc. | FRAGM |
|  | STER0266 | HAD superfamily hydrolase, trunc. | FS |
|  | STER0385 | Conserved hypothetical protein, trunc. | FS |
|  | STER0403 | Acetoin utilization protein,trunc. | FS |
|  | STER0645 | Oxidoreductase, trunc. | FS |
|  | STER0746 | Short chain dehydrogenase/reductase family oxidoreductase, trunc. | FS |
|  | STER0910 | Zn-dependent alcohol dehydrogenase, trunc. | FS |
|  | STER0951 | Putative cell surface hydrolase, trunc. | FS |
|  | STER0996 | Putative HAD superfamily hydrolase, trunc. | FS |
|  | STER1052 | HAD superfamily hydrolase, trunc. | FS |
|  | STER1098 | Putative permease, trunc. | FS |
|  | STER1109 | Putative acetyltransferase, trunc. | FS |
|  | STER1287 | Putative ABC transporter ATPase, trunc. | FS |
|  | STER1354 | Similar to coenzyme PQQ synthesis protein, trunc. | FRAGM |
|  | STER1374 | Putative zinc metalloprotease, trunc. | FS |
|  | STER1377 | ABC transporter ATPase, trunc. | FS |
|  | STER1459 | CAAX amino terminal protease, trunc. | FS |
|  | STER1631 | Probable CAAX amino terminal protease family protein, trunc. | FRAGM |
|  | STER1679 | Putative membrane protein, trunc. | FS |
|  | STER1775 | Putative CoA binding protein, trunc. | FRAGM |
|  | STER1805 | Putative NADPH-dependent FMN reductase, trunc. | FS |
|  | STER1806 | Putative NADPH-dependent FMN reductase, trunc. | FS |
|  | STER1831 | Putative CAAX amino protease family protein, trunc. | FS |
|  | STER1924 | Putative oxidoreductase, trunc. | FRAGM? |
| **[S] Function unknown** | | |  |
|  | STER0063 | Hypothetical protein, trunc. | FS |
|  | STER0119 | Conserved hypothetical protein, trunc. | FS |
|  | STER0125 | Hypothetical protein, trunc. | FS |
|  | STER0134 | Similar to lantibiotic exporter, trunc. | FS |
|  | STER0135 | Hypothetical protein, trunc. | FRAGM |
|  | STER0140 | Transposase, trunc. | FRAGM |
|  | STER0141 | Putative glutamate/aspartate-proton symporter, trunc. | FRAGM |
|  | STER0167 | Conserved membrane protein, trunc. | FRAGM |
|  | STER0202 | 1,6-alpha-glucanhydrolase (dextranase), trunc. | FS |
|  | STER0257 | Conserved hypothetical protein, trunc. | FRAGM |
|  | STER0321 | Urease cluster protein, trunc. | FS |
|  | STER0360 | Conserved hypothetical protein, trunc. | FS |
|  | STER0454 | Conserved hypothetical protein, trunc. | FS |
|  | STER0459 | DegV family protein, trunc. | FS |
|  | STER0538 | Conserved hypothetical protein, trunc. | FS |
|  | STER0564 | Probable macrolide ABC transporter, permease component, trunc. | FS |
|  | STER0614 | Conserved hypothetical protein, trunc. | FS |
|  | STER0619 | Hypothetical protein, trunc. | FRAGM |
|  | STER0681 | Conserved hypothetical protein, trunc. | FS |
|  | STER0704 | Conserved hypothetical protein, trunc. | FS |
|  | STER0744 | Conserved hypothetical protein, trunc. | FS |
|  | STER0825 | Putative phage-related protein, trunc. | FS |
|  | STER0892 | Putative type I R/M system restriction subunit, trunc. | FRAGM |
|  | STER0979 | Hypothetical protein, trunc. | FS |
|  | STER0982 | Amidase, trunc. | FS |
|  | STER2007 | Hypothetical protein, trunc. | FRAGM |
|  | STER0999 | Conserved hypothetical protein, trunc. | FS |
|  | STER1015 | Hypothetical protein, trunc. | FRAGM |
|  | STER1038 | Hypothetical protein, trunc. | FS |
|  | STER1056 | Hypothetical protein, trunc. | FRAGM |
|  | STER1076 | Hypothetical protein, trunc. | FRAGM |
|  | STER1081 | Probable glucan-binding protein, trunc. | FS |
|  | STER1127 | Hypothetical protein, trunc. | FS |
|  | STER1128 | Conserved hypothetical protein, trunc. | FRAGM |
|  | STER1130 | Putative transmembrane protein, trunc. | FS |
|  | STER1218 | Hypothetical protein, trunc. | FRAGM |
|  | STER1240 | Hypothetical protein, trunc. | FRAGM |
|  | STER1251 | Transposase, trunc. | FS |
|  | STER1284 | Hypothetical protein, trunc. | FRAGM |
|  | STER1336 | LPXTG cell wall surface anchor protein, trunc. | FS |
|  | STER1341 | Hypothetical protein, trunc. | FS |
|  | STER1342 | Hypothetical protein, trunc. | FS |
|  | STER1349 | Hypothetical protein, trunc. | FRAGM |
|  | STER1351 | Hypothetical protein, trunc. | FS |
|  | STER1353 | Hypothetical protein, trunc. | FRAGM |
|  | STER1362 | Conserved hypothetical protein, trunc. | FS |
|  | STER1363 | Conserved hypothetical protein, trunc. | FRAGM |
|  | STER1468 | Conserved hypothetical protein, trunc. | FS |
|  | STER1511 | Transposase, trunc. | FS |
|  | STER1537 | Conserved hypothetical protein, trunc. | FS |
|  | STER1588 | Conserved hypothetical protein, trunc. | FS |
|  | STER1604 | Conserved hypothetical protein, trunc. | FS |
|  | STER1626 | Probable plasmid mobilization protein, trunc. | FRAGM |
|  | STER1658 | Hypothetical protein, trunc. | FRAGM |
|  | STER1671 | Hypothetical protein, trunc. | FS |
|  | STER1687 | Hypothetical protein, trunc. | FS |
|  | STER1706 | Hypothetical protein, trunc. | FS |
|  | STER1734 | Conserved hypothetical protein, trunc. | FS |
|  | STER1802 | Conserved hypothetical protein, trunc. | FRAGM |
|  | STER1815 | Putative HAD family hydrolase, trunc. | FS |
|  | STER1872 | Hypothetical protein, trunc. | FRAGM |
|  | STER1929 | Putative replication initiator protein, trunc. | FS |
|  | STER1957 | Putative membrane protein, trunc. |  |
| **[T] Signal transduction mechanisms** | | |  |
|  | STER0355 | Sensor histidine kinase, trunc. | FS |
|  | STER0473 | Sensor histidine kinase, trunc. | FRAGM |
| (TK) | STER0838 | Response regulator, trunc. | FS |
|  | STER0839 | Sensor histidine kinase, trunc. | FS |
| (TK) | STER1335 | Two-component response regulator, trunc. | FS |
|  | STER1388 | Sensor histidine kinase, trunc. | FS |
| **[V] Defense mechanism** | | |  |
|  | STER0102 | Putative bacteriocin self-immunity protein, trunc. | FS |
|  | STER0318 | ABC bacteriocin/lantibiotic exporter, trunc. | FS |
|  | STER0571 | Putative antimicrobial peptide ABC transporter ATPase, trunc. | FS |
|  | STER0587 | Putative multidrug ABC transporter permease, trunc. | FRAGM |
|  | STER0884 | Type I R/M system restriction subunit, trunc. | FRAGM |
|  | STER0889 | Type I R/M system specificity subunit, trunc | FS |
|  | STER0890 | Type I R/M system methyltransferase subunit, trunc. | FRAGM |
|  | STER0893 | Putative type I R/M system methyltransferase subunit, trunc. | FRAGM |
|  | STER0943 | Putative macrolide efflux ABC transporter permease, trunc. | FRAGM |
|  | STER0959 | MATE efflux family protein DinF, trunc. | FS |
|  | STER1303 | ABC transporter ATPase, trunc. | FRAGM |
|  | STER1352 | Putative Na+-driven multidrug efflux pump, trunc. | FRAGM |
|  | STER1378 | ABC transporter ATPase, trunc. | FS |
|  | STER1625 | Type I R/M system specificity subunit, trunc. | FRAGM |
|  | STER1719 | ABC transporter ATPase component, trunc. | FS |
| ***Phage-related gene*** | | | |
| Phage | STER0574 | Surface-associated protein CshA precursor, trunc. | FRAGM |
| Phage | STER0755 | Similar to streptococcal phage DNase1, trunc. | FS |
| Phage_M | STER1031 | Putative N-acetylmuramidase/endolysin, trunc. | FS |
| Phage | STER1158 | Surface-associated protein CshA precursor, trunc. | FS |

^a^ FRAGM, truncated, FS, frameshift
