# Supplementary material for: Specialized adaptation of a lactic acid bacterium to the milk environment: the comparative genomics of Streptococcus thermophilus LMD-9
Source: Microb Cell Fact. 2011 Aug 30;10(Suppl 1):S22. doi: 10.1186/1475-2859-10-S1-S22 (PMC3231929; doi:10.1186/1475-2859-10-S1-S22)
Supplement: Additional file 3 — S. thermophilus LMD-9 specific gene regions that are absent in both CNRZ1066 and LMG 18311 strains [file 1475-2859-10-S1-S22-S3.doc]

Additional file 3. *S. thermophilus* LMD-9 specific gene regions that are absent in both CNRZ1066 and LMG 18311 strains

| Region (R) | Size (kb) | Associated ORFs^a^ | Putative function | % GC^b^ | Associated mobile element | Organism with closest ortholog (% amino acid identity) |
| --- | --- | --- | --- | --- | --- | --- |
| 1 | 1.8 | STER0131* | Hypothetical protein | 32.0 | Transposase | *Lactococcus lactis* (44%) |
|  |  |  |  |  |  |  |
| 2 | 10.2 | ψSTER0140* | Transposase, trunc. |  | Transposase | *S. thermophilus* (96%) |
|  |  | ψSTER0141* | Putative glutamate/aspartate-proton symporter, trunc. |  |  | *S. thermophilus* CNRZ302 (96%) |
|  |  | STER0142* | Putative dipeptide/oligopeptide ABC transporter, substrate-binding protein | 36.8 |  | *Bifidobacterium bifidum* (51%) |
|  |  | STER0143* | Putative dipeptide/oligopeptide ABC transporter, permease component | 39.4 |  | *Bifidobacterium angulatum* (65%) |
|  |  | STER0144* | Putative dipeptide/oligopeptide ABC transporter, permease component | 41.6 |  | *Bifidobacterium bifidum* (65%) |
|  |  | STER0145* | Putative dipeptide/oligopeptide ABC transporter, ATPase component | 40.3 |  | *Xylanimonas cellulosilytica* (58%) |
|  |  | ψSTER0146* | Putative bacteriocin ABC exporter accessory protein, trunc. |  |  | *Enterococcus faecium* (54%) |
|  |  | STER0147 | IS5 family transposase | 35.9 |  | *S. thermophilus* CNRZ368 (97%) |
|  |  | STER0149* | Glycosyltransferase | 34.8 |  | *Enterococcus faecalis* (90%) |
|  |  | STER0150* | UDP-glucose 6-dehydrogenase | 37.0 |  | *E. faecalis* (90%) |
|  |  |  |  |  |  |  |
| 3 | 1.2 | ψSTER0360 | Conserved hypothetical protein, trunc. |  | Transposase | *S. salivarius* (58%) |
|  |  |  |  |  |  |  |
| 4 | 7.9 | ψSTER0570 | Putative transposase, trunc. |  | Transposase | *Ruminococcus gnavus* (69%) |
|  |  | ψSTER0571 | Putative antimicrobial peptide ABC transporter ATPase, trunc. |  |  | *Leuconostoc mesenteroides* (96%) |
|  |  | STER0572 | Probable antimicrobial peptide ABC transporter permease | 33.1 |  | *L. mesenteroides* (97%) |
|  |  | STER0573 | Hypothetical protein | 33.3 |  | none |
|  |  | ψSTER0574 | Similar to surface-associated protein CshA precursor, trunc. |  |  | *Gemella haemolysans* (78%) |
|  |  | STER0576 | Mucus-binding domain protein | 38.3 |  | *Leuconostoc citreum* (68%) |
|  |  |  |  |  |  |  |
| 5 | 0.8 | STER0631 | Putative transposase IS1167 | 41.3 | Transposase | *S. thermophilus* RD678-7(0) (99%) |
|  |  | STER0656 | Putative dTDP-4-dehydrorhamnose 3,5-epimerase | 36.1 |  | *Lactobacillus rhamnosus* (64%) |
|  |  |  |  |  |  |  |
| 6 | 2.5 | STER0731 | Putative cytosine-C5 specific DNA methylase | 33.3 |  | *Staphylococcus hominis* (78%) |
|  |  | STER0732 | Hypothetical protein | 33.6 |  | *Leptotrichia hofstadii* (61%) |
|  |  |  |  |  |  |  |
| 7 | 4.0 | STER0748 | Hypothetical protein | **28.5** |  | *Mollicutes bacterium* (27%) |
|  |  | STER0749 | Hypothetical protein | **29.7** |  | none |
|  |  | STER0750 | Putative type I R/M system specificity protein, HsdS | 34.9 |  | *E. faecalis* (38%) |
|  |  | STER0751 | Hypothetical protein | **30.6** |  | *Anaerococcus hydrogenalis* (32%) |
|  |  |  |  |  |  |  |
| 8 | 3.7 | STER0810 | Similar to phage protein | 35.9 |  | *S. pyogenes* (71%) |
|  |  | STER0811* | Putative transposase | 31.9 |  | *L. lactis* (93%) |
|  |  | ψSTER0812* | Putative integrase, trunc. |  |  | *L. lactis* (88%) |
|  |  | STER0813 | Putative phage-related protein | 35.0 |  | *Lactococcus* phage (85%) |
|  |  | STER0814* | Putative phage-related protein | **30.4** |  | *Lactococcus* phage (96%) |
|  |  | STER0815 | Hypothetical protein | 42.4 |  | none |
|  |  |  |  |  |  |  |
| 9 | 8.9 | ψSTER0843 | Spermidine/putrescine ABC transporter permease component, trunc. |  | Transposase | *S. suis* (95%) |
|  |  | STER0844 | Spermidine/putrescine ABC transporter substrate-binding protein | 38.5 |  | *S. suis* (92%) |
|  |  | STER0845 | Chloride channel protein EriC | 40.2 |  | *S. suis* (92%) |
|  |  | STER0846 | Cell envelope proteinase PrtS | 41.5 |  | *S. suis* (95%) |
|  |  | STER0849 | Putative IS1167 transposase | 41.4 |  | *S. suis* (83%) |
|  |  |  |  |  |  |  |
| 10 | 2.5 | STER0961 | Hypothetical protein | **28.5** |  | none |
|  |  | STER0962 | MutT/nudix family protein | 41.2 |  | *Streptococcus* sp. 143 (77%) |
|  |  | STER0963 | Hypothetical protein | 40.6 |  | *Streptococcus* sp. 143 (94%) |
|  |  | STER0964 | Similar to phage protein | 36.1 |  | *S. equi* (35/58; 60%) |
|  |  |  |  |  |  |  |
| 11 | 10.5 | STER1057* | PST family polysaccharide transporter EpsU | **29.7** | Transposase | *L. lactis* (98%) |
|  |  | ψSTER1058 | Transposase, trunc. |  |  | *L. lactis* pNP40 (95%) |
|  |  | STER1059* | Putative glycosyltransferase family 2 | **30.0** |  | *Clostridium beijerinckii* (43%) |
|  |  | STER1060* | Putative polysaccharide polymerase | **26.4** |  | *S. pneumoniae* (25%) |
|  |  | STER1061* | Putative glycosyltransferase family 1 | 33.0 |  | *L. gasseri* (49%) |
|  |  | STER1062* | Glycosyltransferase | **30.4** |  | *S. thermophilus* (44%) |
|  |  | STER1063 | Glycosyltransferase | 32.9 |  | *Eubacterium rectale* (67%) |
|  |  | STER1064 | Glycosyltransferase | 40.7 |  | *Ruminococcus obeum* (80%) |
|  |  | STER1065 | Glycosyltransferase | 39.2 |  | *Eubacterium rectale* (90%) |
|  |  | ψSTER1066* | Putative rhamnosyl transferase, trunc. |  |  | *S. infantarius* (89%) |
|  |  |  |  |  |  |  |
| 12 | 8.7 | ψSTER1127 | Hypothetical protein, trunc. |  |  | *S. salivarius* (94%) |
|  |  | ψSTER1128 | Similar to spore coat protein H CotH, trunc. |  |  | *S. salivarius* (95%) |
|  |  | ψSTER1130 | Putative transmembrane protein, trunc. |  |  | *S. salivarius* (96%) |
|  |  | STER1131 | Putative glycosyltransferase | 40.7 |  | *S. salivarius* (98%) |
|  |  | STER1132 | Conserved hypothetical protein | 39.2 |  | *S. salivarius* (97%) |
|  |  | STER1133 | Hypothetical membrane protein | 37.0 |  | *S. salivarius* (87%) |
|  |  | STER1134 | Hypothetical protein | 36.0 |  | *S. salivarius* (83%) |
|  |  |  |  |  |  |  |
| 13 | 0.1 | STER1162 | Putative transposase | 41.4 | Transposase | *S. thermophilus* RD678-7(0) (100%) |
|  |  |  |  |  |  |  |
| 14 | 9.6 | STER1196 | Putative IS4 family transposase | 40.6 | Transposase | *S. suis* (84%) |
|  |  | STER1198 | Phosphoribosyl-ATP pyrophosphatase | 40.6 |  | *S. salivarius* (97%) |
|  |  | STER1199 | Phosphoribosyl-AMP cyclohydrolase | 42.5 |  | *S. salivarius* (97%) |
|  |  | STER1200* | Imidazoleglycerol-phosphate synthase | 46.4 |  | *S. salivarius* (98%) |
|  |  | STER1201* | 1-(5-phosphoribosyl)-5-[(5-phosphoribosylamino)methylideneamino] imidazole-4-carboxamide isomerase | 45.0 |  | *S. salivarius* (92%) |
|  |  | STER1202 | Imidazole glycerol phosphate synthase, glutamine amidotransferase subunit | 42.5 |  | *S. salivarius* (93%) |
|  |  | STER1203 | Imidazoleglycerol-phosphate dehydratase | 41.4 |  | *S. salivarius* (98%) |
|  |  | STER1204 | Histidinol dehydrogenase | 43.0 |  | *S. salivarius* (95%) |
|  |  | STER1205 | ATP phosphoribosyltransferase catalytic subunit | 39.6 |  | *S. salivarius* (95%) |
|  |  | STER1206 | ATP phosphoribosyltransferase regulatory subunit | 42.5 |  | *S. salivarius* (93%) |
|  |  | STER1207 | Histidinol-phosphate aminotransferase | 41.1 |  | *S. salivarius* (94%) |
|  |  | STER1208 | Hypothetical protein | 32.2 |  | none |
|  |  |  |  |  |  |  |
| 15 | 9.2 | STER1296* | Major facilitator superfamily permease | **27.5** |  | *S. gordonii* (44%) |
|  |  | STER1297* | Probable oligoendopeptidase F | **25.5** |  | none |
|  |  | STER1298* | Hypothetical protein | **28.4** |  | none |
|  |  | STER1299* | Putative MutR transcriptional regulator | **27.2** |  | *S. pneumoniae* (43%) |
|  |  | STER1300* | Putative MutT/NudiX family protein | **29.4** |  | *S. infantarius* (63%) |
|  |  | STER1301* | Hypothetical protein | **23.5** |  | none |
|  |  | STER1302 | Hypothetical protein | 37.6 |  | *S. sanguinis* (93%) |
|  |  | ψSTER1303 | ABC transporter ATPase component, trunc. |  |  | *S. salivarius* (91%) |
|  |  | STER1304 | Hypothetical protein | 40.0 |  | *S. salivarius* (96%) |
|  |  | STER1305 | Hypothetical protein | 40.2 |  | *S. salivarius* (98%) |
|  |  | STER1306 | Predicted Fe-S-cluster oxidoreductase | 42.1 |  | *S. salivarius* (99%) |
|  |  |  |  |  |  |  |
| 16 | 2.6 | STER1347 | Peptide ABC transporter permease | 33.0 | Transposase | *L. mesenteroides* (97%) |
|  |  | STER1348 | Peptide ABC transporter ATPase | 33.5 |  | *L. mesenteroides* (96%) |
|  |  | ψSTER1349 | Hypothetical protein, trunc. |  |  | none |
|  |  |  |  |  |  |  |
| 17 | 5.6 | ψSTER1352* | Putative Na+-driven multidrug efflux pump, trunc. |  |  | *S. equi* (77%) |
|  |  | ψSTER1353 | Hypothetical protein, trunc. |  |  | *S. equi* (86%) |
|  |  | ψSTER1354 | Similar to coenzyme PQQ synthesis protein, trunc. |  |  | *S. equi* (92%) |
|  |  | STER1355* | Permease of the major facilitator superfamily | **25.5** |  | *S. gallalolyticus* (51%) |
|  |  | STER1356* | Radical SAM domain-containing protein | 31.4 |  | *L. lactis* (54%) |
|  |  | STER1357 | Cyclic peptide Pep1357C | 47.3 |  | none |
|  |  | STER1358* | Putative Rgg family transcriptional regulator | 30.7 |  | *S. equi* (85%) |
|  |  | STER2008 | Short hydrophobic peptide (SHP)^†^ | 34.8 |  | none |
|  |  |  |  |  |  |  |
| 18 | 1.0 | ψSTER1362 | Conserved hypothetical protein, trunc. |  | Transposase | *S. pneumoniae* (96%) |
|  |  | ψSTER1363 | Conserved hypothetical protein, trunc. |  |  | *S. pneumoniae* (89%) |
|  |  |  |  |  |  |  |
| 19 | 1.3 | STER1412* | Transposase | 37.8 | Transposase | *L. lactis* (100%) |
|  |  | STER1413* | Transposase | 38.5 |  | *L. lactis* (100%) |
|  |  |  |  |  |  |  |
| 20 | 3.0 | STER1438 | Glycosyltransferase | 34.7 |  | *S. salivarius* (96%) |
|  |  | STER1439 | Conserved hypothetical protein | **29.1** |  | *S. salivarius* (97%) |
|  |  | STER1440 | Putative glycosyltransferase involved in cell wall biogenesis | 37 |  | *S. salivarius* (98%) |
|  |  | STER1441 | Putative glycosyltransferase involved in cell wall biogenesis | **30.4** |  | *S. salivarius* (95%) |
|  |  |  |  |  |  |  |
| 21 | 1.3 | STER1445* | Conserved hypothetical protein | 31.1 |  | *Enterococcus casseliflavus* (38%) |
|  |  |  |  |  |  |  |
| 22 | 7.1 | STER1474* | CRISPR-associated protein, Csn2 family | **29.0** |  | *S. mutans* (57%) |
|  |  | STER1475* | CRISPR-associated protein Cas2 | **30.4** |  | *S. agalactiae* (87%) |
|  |  | STER1476* | CRISPR-associated protein Cas1 | 31.7 |  | *S. agalactiae* (80%) |
|  |  | STER1477* | CRISPR-associated endonuclease, Csn1 family | 31.5 |  | *S. mutans* (60%) |
|  |  |  |  |  |  |  |
| 23 | 1.4 | STER1639 | Hypothetical protein | 40.6 |  | none |
|  |  | STER1640 | Hypothetical protein | 32.6 |  | none |
|  |  | STER1641 | Putative bacteriocin | **49.5** |  | *S. salivarius* (58%) |
|  |  | STER1647 | Hypothetical protein | 39.8 |  | none |
|  |  | STER1648 | Hypothetical protein | **51.4** |  | none |
|  |  |  |  |  |  |  |
| 24 | 4.4 | STER1693* | Putative transcriptional regulator, XRE family | **30.5** | Transposase | *S. pneumoniae* (63%) |
|  |  | STER1695 | Putative transcriptional regulator | 43.2 |  | *S. salivarius* (100%) |
|  |  | STER1696 | Conserved hypothetical protein | 38.6 |  | *S. salivarius* (97%) |
|  |  | STER1697 | Hypothetical protein | 41.4 |  | *S. salivarius* (79%) |
|  |  | STER1698* | Abortive infection phage resistance protein | 29.0 |  | *S. pyogenes* (40%) |
|  |  | STER1699 | Hypothetical protein | 31.3 |  | *S. salivarius* (90%) |
|  |  |  |  |  |  |  |
| 25 | 1.0 | ψSTER1795 | Oligopeptide ABC transporter substrate-binding protein, trunc. |  |  | *S. salivarius* (87%) |
|  |  | STER1796 | Hypothetical protein | 26.3 |  | none |
|  |  |  |  |  |  |  |
| 26 | 2.9 | STER1868 | Hypothetical protein | 42.5 | Transposase | *E. faecalis* (94%) |
|  |  | STER1869 | Conserved hypothetical protein | 38.5 |  | *L. fermentum* (71%) |
|  |  | ψSTER1870 | Conserved hypothetical protein, trunc. |  |  | *E. faecalis* (98%) |
|  |  | ψSTER1871 | Putative phage integrase, trunc. |  |  | *S. thermophilus* CNRZ385 (71%) |
|  |  | ψSTER1872 | Hypothetical protein, trunc. |  |  | *L. lactis* pMRC01 (100%) |
|  |  | STER1873 | Hypothetical protein | 36.4 |  | none |
|  |  | STER1874 | Hypothetical protein | 32.9 |  | none |
|  |  |  |  |  |  |  |
| 27 | 0.9 | STER1926 | Transcriptional regulator | 33.5 |  | *Streptococcus* sp. 2_1_36FAA (96%) |
|  |  |  |  |  |  |  |

^a^ ψ indicates pseudogene

^b^ %GC of ORFs that deviates from the mean %GC content of LMD9 (39.08) for ± 2SD (8.46) are indicated in bold.

* indicates genes that were previously identified by Liu et al. (38) as potentially horizontally acquired by LMD-9.

^†^ This SHP is identified by Ibrahim et al. (15) that is involved in the production of the cyclic peptide Pep1357C encoded by STER1357.
