# Supplementary material for: Specialized adaptation of a lactic acid bacterium to the milk environment: the comparative genomics of Streptococcus thermophilus LMD-9
Source: Microb Cell Fact. 2011 Aug 30;10(Suppl 1):S22. doi: 10.1186/1475-2859-10-S1-S22 (PMC3231929; doi:10.1186/1475-2859-10-S1-S22)
Supplement: Additional file 4 — S. thermophilus-specific genes not present in S. salivarius SK126 draft genome [file 1475-2859-10-S1-S22-S4.doc]

Additional file 4. *S. thermophilus*-specific genes not present in *S. salivarius* SK126 draft genome

| COG^a^ | Locus tag^b^ | Putative gene product | % GC^c^ | Organism with closest orthologs (% amino acid identity) |
| --- | --- | --- | --- | --- |
| [E] Amino acid transport and metabolism | | | | |
|  | STER0322 | Urea transporter | 38.6 | *S. salivarius* 57.I (99%) |
|  | STER0323 | Urea amidohydrolase (urease) gamma subunit UreA | 39.3 | *S. salivarius* 57.I (99%) |
|  | STER0324 | Urea amidohydrolase (urease) beta subunit UreB | 42.6 | *S. salivarius* 57.I (99%) |
|  | STER0325 | Urea amidohydrolase (urease) alpha subunit UreC | 41.4 | *Streptococcus vestibularis* (99%) |
|  | **STER0938** | Putative arginase/agmatinase/formiminoglutamase | **28.2** | *Candidatus Parvarchaeum acidophilus* (29%) |
|  | STER0984 | Dipeptide/tripeptide permease | 44.2 | *Weissella paramesenteroides* (74%) |
|  | **STER1344** | Putative homoserine/threonine efflux protein | 31.4 | *Teredinibacter turnerae* (35%) |
|  |  |  |  |  |
| [G] Carbohydrate transport and metabolism | | | | |
|  | STER0891 | Putative glucose/ribose porter | 35.9 | *Lactobacillus rhamnosus* (91%) |
|  | STER1366 | Beta-galactosidase LacZ | 37.2 | *S. salivarius* ATCC 25975 (94%) |
|  | STER1367 | lactose permease LacS | 38.5 | *S. salivarius* ATCC 25975 (97%) |
|  | STER1368 | aldose 1-epimerase GalM | 35.1 | *S. salivarius* ATCC 25975 (94%) |
|  |  |  |  |  |
| [H] Coenzyme transport and metabolism | | | | |
|  | STER0126 | 2-dehydropantoate 2-reductase | 38.6 | *Streptococcus gallolyticus* (97%) |
|  | **STER0585** | Molybdopterin/thiamine biosynthesis family protein | 33.2 | *E. faecalis (57%)* |
|  |  |  |  |  |
| [J] Translation, ribosomal structure and biogenesis | | | | |
|  | STER1166 | Putative translation factor (SUA5) | 39.7 | *Streptococcus parasanguinis* (93%) |
|  |  |  |  |  |
| [K] Transcription | | | | |
|  | STER0136 | Putative transcriptional regulator | 35.7 | *Streptococcus agalactiae* (71%) |
| (KL) | STER1330 | Type II R/M system restriction subunit | 35.1 | *Catonella morbi* (65%) |
| (KT) | STER0583 | Putative response regulator, LytR/AlgR family | 31.1 | *Streptococcus parasanguinis* (79%) |
|  |  |  |  |  |
| [L] Replication, recombination and repair | | | | |
|  | STER0148 | Transposase | 39.2 | *Leuconostoc mesenteroides* (97%) |
|  | STER0152 | Transposase, trunc. |  | *Enterococcus faecium* (94%) |
|  | STER0367 | IS1191 transposase | 39.0 | *Streptococcus macedonicus* (99%) |
|  | STER0441 | IS1216 transposase, trunc. |  | *E. faecalis* (98%) |
|  | STER0547 | IS3 family transposase orf1 | 39.7 | *Streptococcus equi* (70%) |
|  | STER0840 | Putative ISSth1 transposase (orf1), IS3 family | 40.5 | *Streptococcus equi* (71%) |
|  | STER0847 | Putative ISSth1 transposase (orf1), IS3 family | 41.3 | *Streptococcus equi* (71%) |
|  | STER0883 | Putative IS1191 transposase | 38.9 | *Streptococcus macedonicus* (97%) |
|  | STER0888 | Putative IS1191 transposase | 39.2 | *Streptococcus macedonicus* (98%) |
|  | STER1867 | Transposase | 37.3 | *Enterococcus faecalis* (99%) |
|  | **STER1875** | IS30 family transposase, trunc. |  | *Aerococcus viridans* (58%) |
|  | **STER1879** | Putative transposase | 36.0 | *Selenomonas sputigena* (57%) |
| (LR) | STER0709 | CRISPR-associated endonuclease, Csn1 family | 35.2 | *Streptococcus gordonii* (69%) |
| (LR) | STER0710 | CRISPR-associated protein, Cas1 family | 37.4 | *Streptococcus gordonii* (83%) |
| (LR) | STER0970 | CRISPR-associated protein Cas1 | 37.0 | *Streptococcus sanguinis* (52%) |
| (LR) | STER0975 | CRISPR-associated Csm3 family protein | 36.8 | *Streptococcus* sp. M143 (80%) |
| (LR) | STER0976 | CRISPR-associated Csm4 family protein | 37.1 | *Streptococcus* sp. M143 (70%) |
| (LR) | STER0977 | CRISPR-associated Csm5 family protein | 34.3 | *Streptococcus* sp. M143 (73%) |
|  |  |  |  |  |
| [M] Cell wall, membrane, envelope biogenesis | | | | |
|  | STER0187 | cyclopropane-fatty-acyl-phospholipid synthase Cfa | 44.3 | *W. paramesenteroides* (78%) |
|  |  |  |  |  |
| [O] Posttranslational modification, protein turnover, chaperones | | | | |
|  | STER0326 | Urease accessory protein UreE | 37.3 | *S. vestibularis* (99%) |
|  | STER0327 | Urease accessory protein UreF | 40.1 | *S. salivarius 57.*I (97%) |
|  | STER0329 | Urease accessory protein UreH | 37.3 | *S. salivarius* 57.I (96%) |
| (OK) | STER0328 | Urease accessory protein | 39.0 | *Streptococcus infantarius* (93%) |
|  |  |  |  |  |
| [P] Inorganic ion transport and metabolism | | | | |
|  | STER0330 | Cobalt transport protein CbiM | 41.4 | *S. salivarius 57.*I (99%) |
|  | STER0331 | Cobalt ABC transporter, permease component CbiQ | 39.9 | *S. salivarius 57.*I (97%) |
|  | STER1027 | Iron compound ABC transporter permease component | 37.8 | *S. gallolyticus* (54%) |
|  |  |  |  |  |
| [R] General function prediction only | | | | |
|  | **STER0935** | Hypothetical protein | **30.0** | None |
|  | **STER0937** | Probable Fe-S oxidoreductase | **30.6** | *Pelotomaculum thermopropionicum* (27%) |
|  | STER1285 | Putative ABC transporter permease component | 38.6 | *Streptococcus* sp. 2_1_36FAA (98%) |
|  | STER1286 | Putative ABC transporter permease component | 40.1 | *S. pneumoniae* (72%) |
|  | STER1627 | ABC transporter permease component | 46.3 | *L. lactis* (98%) |
|  | STER1823 | Putative membrane protein | 39.7 | *S. gallolyticus* (76%) |
|  | **STER1924** | Putative oxidoreductase, trunc. |  | *Clostridium perfringens* (31%) |
| (RTKL) | **STER1920** | Probable serine/threonine protein kinase | **25.0** | *Bacillus cereus* (32%) |
|  |  |  |  |  |
| [S] Function unknown | | | | |
|  | STER0125 | Hypothetical protein, trunc. |  | *S. gallolyticus* (94%) |
|  | **STER0232** | Major facilitator superfamily permease | 42.3 | *Gemella haemolysans* (34%) |
|  | STER0597 | Conserved hypothetical protein | 31.7 | *Streptococcus agalactiae* (56%) |
|  | **STER0704** | Conserved hypothetical protein, trunc. |  | *Enterococcus* sp. 7L76 (61%) |
|  | STER0712 | Conserved hypothetical protein | 32.2 | *S. gordonii* (42%) |
|  | STER0979 | Hypothetical protein, trunc. |  | *Streptococcus* sp. M143 (41%) |
|  | STER1331 | Type II R/M system methylase subunit | 36.9 | *Streptococcus agalactiae* (65%) |
|  | STER1338 | Conserved hypothetical protein | 35.8 | *Streptococcus mitis* (65%) |
|  | **STER1345** | Hypothetical protein | 36.4 | None |
|  | **STER1634** | Conserved hypothetical protein | 33.1 | None |
|  | STER1718 | Hypothetical protein | 35.4 | *S. mutans* (31%) |
|  | STER1958 | Conserved hypothetical protein | 32.5 | *S. infantarius* (88%) |
|  | STER1960 | Conserved hypothetical protein | **29.8** | *S. infantarius* (97%) |
|  | STER1962 | Conserved hypothetical protein | 33.9 | *S. infantarius* (84%) |
|  |  |  |  |  |
| [T] Signal transduction mechanisms | | | | |
|  | STER0582 | Sensor histidine kinase | 31.4 | *S. parasanguinis* (72%) |
|  |  |  |  |  |
| [V] Defense mechanisms | | | | |
|  | STER1054 | Hypothetical protein | 34.9 | *S. thermophilus* NCFB 2393 (97%) |
|  | STER1719 | ABC transporter ATPase component, trunc. |  | *Streptococcus ratti (43%)* |

^a^ ORFs-encoded proteins belonging to more than one COG category are indicated in bracket.

^b^ ORFs not present in all other streptococci are indicated in bold.

^c^ %GC of ORFs that deviates from the mean %GC content of LMD9 (39.08) for ± 2SD (8.46) are indicated in bold.
