# Supplementary material for: Specialized adaptation of a lactic acid bacterium to the milk environment: the comparative genomics of Streptococcus thermophilus LMD-9
Source: Microb Cell Fact. 2011 Aug 30;10(Suppl 1):S22. doi: 10.1186/1475-2859-10-S1-S22 (PMC3231929; doi:10.1186/1475-2859-10-S1-S22)
Supplement: Additional file 5 — Unique genes among the sub-clusters of S. pneumoniae strains [file 1475-2859-10-S1-S22-S5.doc]

Additional file 5. Unique genes among the sub-clusters of *S. pneumoniae* strains

| Locus tag | Putative function |
| --- | --- |
| *S. pneumoniae* sub-cluster 1-specific genes | |
| spr0315 | Polysaccharide polymerase Cps2H |
| spr0316 | Group I glycosyltransferase Cps2I |
| spr0317 | Putative polysaccharide transporter Cps2J |
| spr0319 | UDP-galactopyranose mutase Cps2P |
| spr0961 | UDP-N-acetyl-D-mannosaminuronic acid dehydrogenase RffD |
| spr0962 | Conserved hypothetical protein |
| spr0963 | Hypothetical protein |
| spr0964 | Hypothetical protein |
| spr0965 | Hypothetical protein |
| spr0967 | Conserved hypothetical protein |
| spr0968 | Hypothetical protein |
|  |  |
| *S. pneumoniae* sub-cluster 2-specific genes | |
| SP70585_0414 | Tyrosine-protein phosphatase CpsB |
| SP70585_0415 | Capsular polysaccharide biosynthesis protein |
| SP70585_0416 | Tyrosine-protein kinase CpsD |
| SP70585_0606 | Immunity protein BlpX |
| SP70585_0926 | Conserved hypothetical protein |
| SP70585_0927 | Prophage maintenance system killer protein |
| SP70585_1268 | Potassium/ion channel protein |
|  |  |
