# Supplementary material for: Specialized adaptation of a lactic acid bacterium to the milk environment: the comparative genomics of Streptococcus thermophilus LMD-9
Source: Microb Cell Fact. 2011 Aug 30;10(Suppl 1):S22. doi: 10.1186/1475-2859-10-S1-S22 (PMC3231929; doi:10.1186/1475-2859-10-S1-S22)
Supplement: Additional file 6 — Unique genes among the sub-clusters of S. pyogenes strains [file 1475-2859-10-S1-S22-S6.doc]

Additional file 6. Unique genes among the sub-clusters of *S. pyogenes* strains

| Locus tag | Putative function |
| --- | --- |
| *S. pyogenes* sub-cluster SY1(SF370)-specific genes | |
| Spy_0942 | Hypothetical protein |
| Spy_0944 | Putative phage-related protein |
| Spy_0978 | Putative phage-related protein |
| Spy_2147 | Putative phage-related protein |
|  |  |
| *S. pyogenes* sub-cluster SY2-specific genes (excludes SY1) | |
| MGAS10270_Spy0165 | Co-activator of prophage gene expression IbrA |
| MGAS10270_Spy0166 | Co-activator of prophage gene expression IbrB |
| MGAS10270_Spy0911 | Hypothetical protein |
| MGAS10270_Spy0918 | Lantibiotic biosynthesis sensor protein |
| MGAS10270_Spy0921 | Lanthionine synthetase |
| MGAS10270_Spy0922 | Serine/threonine dehydratase |
| MGAS10270_Spy0925 | Lantibiotic transport permease protein |
| MGAS10270_Spy0932 | DNA integration/recombination/inversion protein |
| MGAS10270_Spy1000 | Transcriptional regulators, LysR family |
| MGAS10270_Spy1004 | D-2-hydroxyacid dehydrogenase |
| MGAS10270_Spy1005 | Hypothetical protein |
| MGAS10270_Spy1006 | Hypothetical protein |
| MGAS10270_Spy1366 | Hypothetical protein |
| MGAS10270_Spy1367 | Hypothetical protein |
| MGAS10270_Spy1368 | Hypothetical protein |
| MGAS10270_Spy1369 | Hypothetical cytosolic protein |
| MGAS10270_Spy1370 | Hypothetical protein |
| MGAS10270_Spy1371 | Hypothetical protein |
| MGAS10270_Spy1372 | ATP-dependent RNA helicase |
| MGAS10270_Spy1797 | Fibronectin-binding protein |
| MGAS10270_Spy1798 | Serum opacity factor |
|  |  |
| *S. pyogenes* sub-cluster SY3-specific genes | |
| SpyM50471 | Putative integrase |
| SpyM50529 | Putative phage holin protein |
| SpyM50565 | Hypothetical protein |
| SpyM50566 | Putative oxidoreductase |
| SpyM50567 | CorA-like Mg2+ transporter protein |
|  |  |
