# Supplementary material for: Specialized adaptation of a lactic acid bacterium to the milk environment: the comparative genomics of Streptococcus thermophilus LMD-9
Source: Microb Cell Fact. 2011 Aug 30;10(Suppl 1):S22. doi: 10.1186/1475-2859-10-S1-S22 (PMC3231929; doi:10.1186/1475-2859-10-S1-S22)
Supplement: Additional file 7 — ORFs with predicted signal peptide sequence in S. thermophilus LMD-9 [file 1475-2859-10-S1-S22-S7.doc]

Additional file 7. ORFs with predicted signal peptide sequence in *S. thermophilus* LMD-9

| COG Group | Locus tag | | Length (aa) | Putative function | COG gene | LPXTG motif/TAT motif |
| --- | --- | --- | --- | --- | --- | --- |
| [C] Energy production and conversion | | | | | | |
|  | STER0293 | | 93 | Acylphosphatases | AcyP |  |
|  | STER0344 | | 403 | Putative serine/threonine transporter | GltP |  |
|  | STER0515 | | 66 | F_0_F_1_ ATP synthase subunit C | AtpE |  |
|  | STER0516 | | 236 | F_0_F_1_ ATP synthase subunit A | AtpB |  |
| [D] Cell cycle control, cell division, chromosome partitioning | | | | | | |
|  | STER0523 | | 427 | Cell division protein FtsW | FtsW |  |
|  | STER1100 | | 310 | Cell division ABC transporter, permease protein FtsX | FtsX |  |
|  | STER1197 | | 476 | Cell division protein FtsW | FtsW |  |
| [E] Amino acid transport and metabolism | | | | | | |
|  | STER0116 | | 207 | Serine acetyltransferase | CysE |  |
|  | STER0142 | | 552 | Putative dipeptide/oligopeptide ABC transporter, substrate-binding protein | OppA |  |
|  | STER0398 | | 392 | Branched-chain amino acid ABC transporter, substrate-binding protein | LivK |  |
|  | STER0506 | | 429 | Homoserine dehydrogenase | ThrA |  |
|  | STER0627 | | 459 | Amino acid permease | LysP |  |
|  | STER0844 | | 357 | Spermidine/putrescine ABC transporter, substrate-binding protein | PotD |  |
|  | STER0863 | | 394 | Aspartate aminotransferase | - |  |
|  | STER0905 | | 214 | Polar amino acid ABC transporter, permease component | HisM |  |
|  | STER1282 | | 224 | L-serine dehydratase beta subunit | SdaA |  |
|  | STER1315 | | 443 | Branched chain amino acid:Na+ symporter | BrnQ |  |
|  | STER1318 | | 462 | Amino acid permease | PotE |  |
|  | STER1344 | | 206 | Putative homoserine/threonine efflux protein | RhtB |  |
|  | STER1409 | | 656 | Oligopeptide ABC transporter, substrate-binding protein | OppA |  |
|  | STER1411 | | 658 | Oligopeptide ABC transporter, substrate-binding protein | OppA |  |
|  | STER1493 | | 357 | Putrescine/spermidine ABC transporter, substrate-binding protein | PotD |  |
|  | STER1494 | | 260 | Spermidine/putrescine ABC transporter, permease component | PotC |  |
|  | STER1495 | | 265 | Spermidine/putrescine ABC transporter, permease component | PotB |  |
|  | STER1496 | | 385 | Spermidine/putrescine ABC transporter, ATPase component | PotA |  |
|  | STER1550 | | 194 | N-(5'-phosphoribosyl)anthranilate isomerase | TrpF |  |
|  | STER1618 | | 308 | Polar amino acid transporter, permease component | HisM |  |
|  | STER1744 | | 232 | Similar to branched-chain amino acid permease (azaleucine resistance) | AzlC |  |
| (EF) | STER0559 | | 1060 | Carbamoyl phosphate synthase large subunit | CarB |  |
| (EP) | STER0143 | | 353 | Putative dipeptide/oligopeptide ABC transporter, permease component | DppB |  |
| (EP) | STER0144 | | 318 | Putative dipeptide/oligopeptide ABC transporter, permease component | DppC |  |
| (EP) | STER1408 | | 498 | Oligopeptide ABC transporter, permease component | DppB |  |
| (ET) | STER0214 | | 539 | Amino acid ABC transporter, substrate-binding protein | HisJ |  |
| (ET) | STER0333 | | 283 | Putative amino acid ABC transporter, substrate-binding protein | HisJ |  |
| (ET) | STER0339 | | 283 | Polar amino acid ABC transporter, substrate-binding protein | HisJ |  |
| (ET) | STER0907 | | 284 | Polar amino acid ABC transporter, substrate-binding protein | HisJ |  |
| (ET) | STER1118 | | 278 | Amino acid ABC transporter, substrate-binding protein | HisJ |  |
| (ET) | STER1452 | | 265 | Amino acid ABC transporter, substrate-binding protein | HisJ |  |
| (ET) | STER1454 | | 266 | Amino acid ABC transporter, substrate-binding protein | HisJ |  |
| (ET) | STER1455 | | 277 | Amino acid ABC transporter, substrate-binding protein | HisJ |  |
| (ET) | STER1461 | | 737 | Glutamine ABC transporter, substrate-binding/permease component | HisJ |  |
| (ET) | STER1539 | | 285 | Putative polar amino acid ABC transporter, substrate-binding protein | HisJ |  |
| (ET) | STER1619 | | 277 | Polar amino acid transporter, substrate-binding protein | HisJ |  |
| [F] Nucleotide transport and metabolism | | | | | | |
|  | STER0059 | | 167 | [Phosphoribosylaminoimidazole carboxylase catalytic subunit](http://www.ncbi.nlm.nih.gov/entrez/viewer.fcgi?db=protein&val=116627007) | PurE |  |
|  | STER0198 | | 847 | Putative cyclo-nucleotide phosphodiesterase | UshA |  |
| (FE) | STER1425 | | 324 | Ribose-phosphate pyrophosphokinase | PrsA |  |
| [G] Carbohydrate transport and metabolism | | | | | | |
|  | STER0249 | | 585 | ABC transporter substrate-binding protein, trunc. | AraH |  |
|  | STER0371 | | 276 | Mannose PTS EIIC | ptnC |  |
|  | STER0504 | | 278 | Acetylornithine aminotransferase | CDA1 |  |
|  | STER0661 | | 279 | Hypothetical protein | - |  |
|  | STER0891 | | 296 | Putative glucose/ribose porter | GlcU |  |
|  | STER1632 | | 288 | Glycerol uptake facilitator protein | GlpF |  |
|  | STER1652 | | 456 | BlpC ABC transporter |  |  |
| (GEPR) | STER0207 | | 388 | Major facilitator transporter | ProP |  |
| (GEPR) | STER1416 | | 402 | Putative oxalate:formate antiporter | ProP |  |
| (GER) | STER1472 | | 297 | Permease of the drug/metabolite transporter (DMT) superfamily | RhaT |  |
| (GER) | STER1587 | | 305 | Putative permease of the drug/metabolite transporter (DMT) superfamily | RhaT |  |
| [H] Coenzyme transport and metabolism | | | | | | |
|  | STER0660 | | 285 | Methylenetetrahydrofolate dehydrogenase/methenyltetrahydrofolate cyclohydrolase | FolD |  |
| (HE) | STER1489 | | 365 | Phosphoserine aminotransferase | SerC |  |
| [I] Lipid transport and metabolism | | | | | | |
|  | STER0245 | | 265 | Phosphatidate cytidylyltransferase | CdsA |  |
|  | STER0432 | | 309 | Acyl-carrier-protein S-malonyltransferase | FabD |  |
|  | STER1279 | | 484 | Cardiolipin synthetase | Cls |  |
|  | STER1522 | | 252 | 1-acyl-sn-glycerol-3-phosphate acyltransferase | PlsC |  |
|  | STER1986 | | 181 | Phosphatidylglycerophosphate synthase | PgsA |  |
| [J] Translation, ribosomal structure and biogenesis | | | | | | |
|  | STER0005 | | 372 | Translation associated GTPase | - |  |
|  | STER0927 | | 448 | tRNA (uracil-5-)-methyltransferase Gid | Gid |  |
|  | STER1902 | | 115 | 50S ribosomal protein L22 | RplV |  |
| [K] Transcription | | | | | | |
|  | STER0378 | | 409 | Transcriptional regulator | LytR |  |
|  | STER0552 | | 302 | LysR family transcriptional regulator | fhuR |  |
|  | STER1071 | | 487 | Transcriptional activator-exopolysaccharide biosynthesis EpsA | LytR |  |
|  | STER1397 | | 105 | DNA-directed RNA polymerase omega chain | RpoZ |  |
|  | STER1530 | | 288 | Transcriptional regulator, XRE family | HipB |  |
| (KR) | STER1488 | | 186 | Acetyltransferase, GNAT family | WecD |  |
| [L] Replication, recombination and repair | | | | | | |
|  | STER0306 | | 254 | Site-specific tyrosine recombinase XerD-like protein | XerD |  |
|  | STER0923 | | 715 | DNA topoisomerase I | TopA |  |
|  | STER1521 | | 232 | Late competence protein required for DNA binding and uptake | ComEA |  |
|  | STER1740 | | 297 | Ribonuclease HIII | RnhC |  |
| (LK) | STER0007 | | 1169 | Transcription coupling factor | Mfd |  |
| (LR) | STER0975 | | 221 | CRISPR-associated Csm3 family protein |  |  |
| [M] Cell wall, membrane, envelope biogenesis | | | | | | |
|  | STER0040 | | 277 | Cell shape-determining protein MreC | MreC |  |
|  | STER0113 | | 423 | D-alanyl-D-alanine carboxypeptidase | DacC |  |
|  | STER0159 | | 266 | D-alanyl-D-alanine carboxypeptidase | VanY |  |
|  | STER0217 | | 386 | undecaprenyl pyrophosphate phosphatase | Rfe |  |
|  | STER0662 | | 705 | penicillin-binding protein 2B | FtsI |  |
|  | STER0741 | | 316 | Lyzozyme M1 (1,4-beta-N-acetylmuramidase) | Acm |  |
|  | STER0805 | | 423 | Putative D-alanyl-lipoteichoic acid biosynthesis protein DltD | DltD |  |
|  | STER1255 | | 254 | Sortase | SrtA |  |
|  | STER1433 | | 571 | Glycosyltransferase | RfaG |  |
|  | STER1666 | | 756 | Putative penicillin-binding protein 2X | FtsI |  |
|  | STER1810 | | 305 | UDP-glucose pyrophosphorylase | GalU |  |
| [N] Cell motility and secretion | | | | | | |
| (NU) | STER0160 | | 195 | Peptidoglycan hydrolase | FlgJ |  |
| (NU) | STER0533 | | 219 | Putative peptidoglycan hydrolase | FlgJ |  |
| (NU) | STER1839 | | 121 | Putative competence protein | PulG |  |
| [O] Posttranslational modification, protein turnover, chaperones | | | | | | |
|  | STER0014 | | 656 | Putative cell division protein FtsH | HflB |  |
|  | STER0218 | | 257 | ABC transporter ATPase | SufC |  |
|  | STER0757 | | 300 | Heat shock protein HtpX | HtpX |  |
|  | STER0846 | | 1619 | Cell envelope proteinase PrtS | AprE | LPNTG |
|  | STER1150 | | 240 | Putative Zn-dependent protease | - |  |
|  | STER2002 | | 412 | Putative trypsin-like serine endoprotease | DegQ |  |
| (OC) | STER1638 | | 231 | Probable immunity/modification protein, trunc. | TrxA |  |
| (OC) | STER1779 | | 112 | Thioredoxin domain-containing protein | TrxA |  |
| [P] Inorganic ion transport and metabolism | | | | | | |
|  | STER0340 | | 301 | ABC transporter substrate-binding protein | NlpA |  |
|  | STER0347 | | 569 | Putative cobalt ABC transporter, ATPase component | CbiO |  |
|  | STER0348 | | 277 | Putative cobalt ABC transporter, permease component | CbiQ |  |
|  | STER0350 | | 224 | Putative Trk-type K^+^ transport systems, NAD-binding component | TrkA |  |
|  | STER0351 | | 462 | Putative Trk-type K^+^ transport systems, permease component | TrkG |  |
|  | STER0531 | | 412 | Ammonia permease | AmtB |  |
|  | STER0786 | | 421 | Putative Mn^2+^ and Fe^2+^ transporter, NRAMP family | MntH |  |
|  | STER0895 | | 515 | Putative zinc (Zn^2+^) ABC transporter, substrate-binding protein | LraI |  |
|  | STER0947 | | 299 | Putative cation (Co/Zn/Cd) efflux protein | CzcD |  |
|  | STER1006 | | 292 | Phosphate ABC transporter, phosphate-binding protein | PstS |  |
|  | STER1007 | | 305 | Phosphate ABC transporter, permease protein | PstC |  |
|  | STER1022 | | 562 | Putative high-affinity Fe^2+^/Pb^2+^ permease | FTR1 |  |
|  | STER1023 | | 402 | Predicted iron-dependent peroxidase | - | TAT signal sequence and TAT motif |
|  | STER1025 | | 349 | Iron compound ABC uptake transporter, substrate-binding protein | FepB |  |
|  | STER1027 | | 323 | Iron compound ABC transporter, permease component | FepD |  |
|  | STER1028 | | 318 | Iron compound ABC transporter, permease component | FepD |  |
|  | STER1042 | | 399 | Putative cation (Co/Zn/Cd) efflux transporter | MMT1 |  |
|  | STER1145 | | 409 | Putative chloride channel protein | EriC |  |
|  | STER1147 | | 512 | Chloride channel protein | EriC |  |
|  | STER1546 | | 743 | Putative copper-translocating P-type ATPase | ZntA |  |
|  | STER1597 | | 283 | Putative metal ion ABC transporter, substrate-binding protein | NlpA |  |
|  | STER1608 | | 689 | NhaP-type Na^+^/H^+^ and K^+^/H^+^ antiporter | NhaP |  |
|  | STER1623 | | 261 | Carbonic anhydrase | Cah |  |
| [R] General function prediction only | | | | | | |
|  | STER0042 | | 456 | Putative peptidoglycan hydrolase PcsB | - |  |
|  | STER0057 | | 280 | Cell wall protein precursor | - |  |
|  | STER0236 | | 313 | Putative malate permease | - |  |
|  | STER0375 | | 474 | Probable xanthine/uracil permease | - |  |
|  | STER0388 | | 544 | Putative polysaccharide transporter | RfbX |  |
|  | STER0442 | | 533 | Conserved hypothetical protein | - |  |
|  | STER0478 | | 500 | Cell segregation protein | - |  |
|  | STER0511 | | 254 | Conserved hypothetical protein | - |  |
|  | STER0618 | | 226 | Putative hemolysin III | - |  |
|  | STER0721 | | 133 | Conserved hypothetical protein | - |  |
|  | STER0722 | | 147 | Conserved hypothetical protein | GvpP |  |
|  | STER0726 | | 180 | Biotin synthase | BioY |  |
|  | STER0856 | | 357 | Putative ABC transporter, substrate-binding protein | Med |  |
|  | STER0858 | | 356 | ABC transporter, permease component | - |  |
|  | STER0859 | | 319 | ABC transporter, permease component | - |  |
|  | STER1155 | | 334 | ABC transporter, substrate-binding protein | - |  |
|  | STER1322 | | 288 | Conserved hypothetical protein | - |  |
|  | STER1520 | | 747 | DNA internalization-related competence protein ComEC/Rec2 | ComEC |  |
|  | STER1606 | | 462 | Hemolysin-like protein containing CBS domains | TlyC |  |
|  | STER1627 | | 252 | ABC transporter permease component | - |  |
|  | STER1829 | | 221 | CAAX amino terminal protease family membrane protein | - |  |
|  | STER1831 | | 140 | Putative CAAX amino protease family protein, trunc. | - |  |
| [S] Function unknown | | | | | | |
|  | STER0069 | | 152 | Hypothetical protein |  |  |
|  | STER0073 | | 74 | Hypothetical protein |  |  |
|  | STER0119 | | 52 | Conserved hypothetical protein, trunc. |  |  |
|  | STER0210 | | 40 | Hypothetical protein |  | LPNTG |
|  | STER0211 | | 57 | Hypothetical protein |  |  |
|  | STER0232 | | 411 | Major facilitator superfamily permease |  |  |
|  | STER0301 | | 82 | Hypothetical protein | - |  |
|  | STER0310 | | 83 | Conserved hypothetical protein | - |  |
|  | STER0314 | | 186 | Hypothetical protein | - |  |
|  | STER0346 | | 182 | Conserved hypothetical protein | - |  |
|  | STER0464 | | 168 | HdeD family protein | HdeD |  |
|  | STER0509 | | 206 | Conserved hypothetical protein |  |  |
|  | STER0548 | | 88 | Conserved hypothetical protein |  |  |
|  | STER0549 | | 136 | Conserved hypothetical protein |  |  |
|  | STER0576 | | 1010 | MucBP domain protein |  |  |
|  | STER0592 | | 230 | Hypothetical protein | - |  |
|  | STER0614 | | 122 | Conserved hypothetical protein, trunc. |  |  |
|  | STER0630 | | 120 | Conserved hypothetical protein |  |  |
|  | STER0642 | | 79 | Hypothetical protein |  |  |
|  | STER0643 | | 76 | Hypothetical protein |  |  |
|  | STER0659 | | 64 | Hypothetical protein |  |  |
|  | STER0708 | | 204 | Conserved hypothetical protein |  |  |
|  | STER0730 | | 103 | Conserved hypothetical protein |  |  |
|  | STER0734 | | 426 | Conserved hypothetical protein |  |  |
|  | STER0735 | | 160 | Conserved hypothetical protein |  |  |
|  | STER0751 | | 196 | Hypothetical protein |  |  |
|  | STER0759 | | 132 | Conserved hypothetical protein |  |  |
|  | STER0824 | | 69 | Possible phage membrane protein |  |  |
|  | STER0834 | | 192 | Conserved hypothetical protein | - |  |
|  | STER0862 | | 161 | Conserved hypothetical protein | - |  |
|  | STER0916 | | 176 | Conserved hypothetical protein |  |  |
|  | STER0945 | | 71 | Hypothetical protein |  |  |
|  | STER1024 | | 293 | Probable iron transport lipoprotein |  |  |
|  | STER1038 | | 148 | Hypothetical protein, trunc. |  |  |
|  | STER1048 | | 95 | Hypothetical protein |  |  |
|  | STER1095 | | 46 | Hypothetical protein |  |  |
|  | STER1113 | | 83 | Conserved hypothetical protein | - |  |
|  | STER1122 | | 63 | Competence associated protein |  |  |
|  | STER1132 | | 609 | Conserved hypothetical protein |  |  |
|  | STER1133 | | 305 | Hypothetical membrane protein |  |  |
|  | STER1141 | | 59 | Conserved hypothetical protein |  |  |
|  | STER1142 | | 176 | Hypothetical protein |  |  |
|  | STER1149 | | 124 | Hypothetical protein |  |  |
|  | STER1169 | | 94 | Hypothetical protein | - |  |
|  | STER1174 | | 67 | Hypothetical protein |  |  |
|  | STER1176 | | 190 | Conserved hypothetical protein | - |  |
|  | STER1213 | | 215 | Conserved hypothetical protein | - |  |
|  | STER1219 | | 67 | Hypothetical protein |  |  |
|  | STER1231 | | 322 | Conserved hypothetical protein | - |  |
|  | STER1253 | | 323 | Putative secreted protein | - |  |
|  | STER1262 | | 170 | Conserved hypothetical protein | - |  |
|  | STER1263 | | 177 | Hypothetical protein |  |  |
|  | STER1294 | | 60 | Hypothetical protein |  |  |
|  | STER1296 | | 410 | Major facilitator superfamily permease |  |  |
|  | STER1304 | | 226 | Hypothetical protein |  |  |
|  | STER1305 | | 222 | Hypothetical protein |  |  |
|  | STER1334 | | 53 | Hypothetical protein |  |  |
|  | STER1431 | | 493 | Hypothetical membrane protein | - |  |
|  | STER1445 | | 436 | Conserved hypothetical protein | - |  |
|  | STER1458 | | 147 | Hypothetical protein |  |  |
|  | STER1465 | | 43 | Hypothetical protein |  |  |
|  | STER1511 | | 162 | Transposase, trunc. |  |  |
|  | STER1576 | | 212 | Hypothetical protein |  |  |
|  | STER1577 | | 42 | Hypothetical protein |  |  |
|  | STER1588 | | 55 | Conserved hypothetical protein, trunc. |  |  |
|  | STER1616 | | 75 | Hypothetical protein |  |  |
|  | STER1620 | | 78 | Hypothetical protein |  |  |
|  | STER1634 | | 127 | Conserved hypothetical protein |  |  |
|  | STER1639 | | 60 | Hypothetical protein |  |  |
|  | STER1642 | | 134 | Conserved hypothetical protein |  |  |
|  | STER1643 | | 56 | Conserved hypothetical protein |  |  |
|  | STER1645 | | 54 | Hypothetical protein |  |  |
|  | STER1671 | | 129 | Hypothetical protein, trunc. |  |  |
|  | STER1674 | | 47 | Hypothetical protein |  |  |
|  | STER1696 | | 203 | Conserved hypothetical protein |  |  |
|  | STER1697 | | 112 | Hypothetical protein |  |  |
|  | STER1699 | | 82 | Hypothetical protein |  |  |
|  | STER1721 | | 91 | Hypothetical protein |  |  |
|  | STER1725 | | 294 | Conserved hypothetical protein |  |  |
|  | STER1734 | | 59 | Conserved hypothetical protein, trunc. |  |  |
|  | STER1753 | | 168 | Putative membrane protein | - |  |
|  | STER1767 | | 425 | Putative nuclease, RmuC family | - |  |
|  | STER1799 | | 118 | Conserved hypothetical protein |  |  |
|  | STER1801 | | 93 | Hypothetical protein |  |  |
|  | STER1808 | | 152 | Hypothetical membrane protein |  |  |
|  | STER1817 | | 56 | Conserved hypothetical protein |  |  |
|  | STER1826 | | 95 | Conserved hypothetical protein |  |  |
|  | STER1836 | | 106 | Conserved hypothetical protein |  |  |
|  | STER1866 | | 113 | Conserved hypothetical protein |  |  |
|  | STER1947 | | 314 | Conserved hypothetical protein | - |  |
|  | STER1961 | | 57 | Conserved hypothetical protein |  |  |
|  | STER1980 | | 577 | Conserved hypothetical protein |  |  |
|  | STER1981 | | 210 | Transglycosylase-like domain-containing protein |  |  |
|  | STER1982 | | 185 | LysM domain-containing protein |  |  |
| [T] Signal transduction mechanisms | | | | | | |
|  | STER0926 | | 432 | Sensor histidine kinase | BaeS |  |
|  | STER1115 | | 447 | Sensor histidine kinase | VicK |  |
|  | STER1309 | | 337 | Sensor histidine kinase | BaeS |  |
|  | STER1612 | | 359 | Putative protease with PDZ domain | SdrC |  |
|  | STER1912 | | 145 | Putative protein-tyrosine phosphatase | Wzb |  |
|  | STER1977 | | 655 | Putative signaling protein (consist of a modified GGDEF domain and a DHH domain) | - |  |
| (TK) | STER1290 | | 201 | Two-component response regulator | CitB |  |
| [U] Intracellular trafficking, secretion, and vesicular transport | | | | | | |
|  | STER0243 | | 113 | Putative preprotein translocase YajC | YajC |  |
|  | STER0292 | | 306 | OxaA-like protein precursor | YidC |  |
|  | STER0673 | | 79 | Preprotein translocase subunit SecG | SecG |  |
|  | STER1021 | | 243 | Putative sec-independent twin-arginine translocase protein TatC | TatC |  |
|  | STER1096 | | 186 | Signal peptidase I | LepB |  |
|  | STER1789 | | 269 | Putative preprotein translocase subunit YidC | YidC |  |
|  | STER1837 | | 146 | Putative competence protein ComGF | ComGF |  |
|  | STER1840 | | 109 | Putative competence protein ComGC | ComGC |  |
|  | STER1887 | | 432 | Preprotein translocase subunit SecY | SecY |  |
| [V] Defense mechanisms | | | | | | |
|  | STER0011 | | 431 | Beta-lactamase family protein | PenP |  |
|  | STER0471 | | 594 | ABC transporter, ATPase/permease component | MdlB |  |
|  | STER0572 | | 349 | Probable antimicrobial peptide ABC transporter permease | SalY |  |
|  | STER0578 | | 579 | ABC multidrug transporter, ATPase and permease component | MdlB | LPNTG |
|  | STER1313 | | 450 | Na+-driven multidrug efflux pump | NorM |  |
|  | STER1347 | | 349 | Peptide ABC transporter permease | SalY |  |
|  | STER1694 | | 530 | Putative bacteriocin ABC exporter, permease and ATPase components | MdlB |  |
|  | STER1921 | | 523 | Putative multidrug ABC transporter, ATPase and permease component | MdlB |  |
| *Phage-related genes* | | | | | | |
| phage | | STER0813 | 177 | Putative phage-related protein |  |  |
| phage | | STER0822 | 119 | Phage-related protein |  |  |
| phage | | STER1121 | 294 | Competence associated membrane nuclease |  |  |
| phage | | STER1509 | 160 | Predicted membrane protein | - |  |
| phage_J | | STER1470 | 102 | Hypothetical protein | - |  |
| phage_M | | STER1031 | 243 | Putative N-acetylmuramidase/endolysin, trunc. | Acm |  |
