# Supplementary material for: Specialized adaptation of a lactic acid bacterium to the milk environment: the comparative genomics of Streptococcus thermophilus LMD-9
Source: Microb Cell Fact. 2011 Aug 30;10(Suppl 1):S22. doi: 10.1186/1475-2859-10-S1-S22 (PMC3231929; doi:10.1186/1475-2859-10-S1-S22)
Supplement: Additional file 8 — Two-component regulatory systems (2CRS) in S. thermophilus LMD-9 [file 1475-2859-10-S1-S22-S8.doc]

Additional file 8. Two-component regulatory systems (2CRS) in *S. thermophilus* LMD-9

| 2CRS | Locus tag | Best blastP hit | Organism | % aa identity | Family |
| --- | --- | --- | --- | --- | --- |
| 1 | STER0354 | response regulator | *S. salivarius* SK126 | 95% | OmpR |
|  |  | CovR | *S. pyogenes* | 81% |  |
|  | ψSTER0355 | sensor histidine kinase | *S. salivarius* SK126 | 91% |  |
|  |  |  |  |  |  |
| 2 | ψSTER0473 | sensor histidine kinase | *S. salivarius* SK126 | 90% |  |
|  |  |  |  |  |  |
| 3 | STER0583 | response regulator SEQ 2172 | *S. equi* | 40% | LytR/AlgR |
|  | STER0582 | sensor histidine kinase SEQ 2173 | *S. equi* | 29% |  |
|  |  |  |  |  |  |
| 4 | ψSTER0838 | transcriptional activator protein CopR | *S. salivarius* SK126 | 93% | CiaR |
|  | ψSTER0839 | sensor protein CiaH | *S. salivarius* SK126 | 90% |  |
|  |  |  |  |  |  |
| 5 | STER0925 | DNA-binding response regulator, OmpR family | *S. salivarius* SK126 | 94% | OmpR |
|  | STER0926 | sensor histidine kinase | *S. salivarius* SK126 | 89% |  |
|  |  |  |  |  |  |
| 6 | STER1115 | sensor protein YycG | *S. salivarius* SK126 | 95% |  |
|  | STER1116 | transcriptional regulatory protein | *S. salivarius* SK126 | 98% | OmpR |
|  |  |  |  |  |  |
| 7 | STER1290 | DNA-binding response regulator | *S. salivarius* SK126 | 99% | LuxR-like |
|  | STER1291 | signal transduction histidine kinase | *S. salivarius* SK126 | 96% |  |
|  |  |  |  |  |  |
| 8 | STER1309 | signal transduction histidine kinase | *S. salivarius* SK126 | 97% |  |
|  | STER1310 | response regulator | *S. salivarius* SK126 | 96% | OmpR |
|  |  |  |  |  |  |
| 9 | ψSTER1335 | response regulator SaeR | *S. salivarius* SK126 | 89% |  |
|  |  |  |  |  |  |
| 10 | STER1387 | DNA-binding response regulator | *S. salivarius* SK126 | 99% |  |
|  | ψSTER1388 | sensor histidine kinase | S. *salivarius* SK126 | 96% |  |
|  |  |  |  |  |  |
| 11 | STER1649 | response regulator | *S. salivarius* SK126 | 40% | LytR/AlgR |
|  | STER1650 | histidine kinase | *S.* *salivarius* SK126 | 47% |  |
