# Supplementary material for: Specialized adaptation of a lactic acid bacterium to the milk environment: the comparative genomics of Streptococcus thermophilus LMD-9
Source: Microb Cell Fact. 2011 Aug 30;10(Suppl 1):S22. doi: 10.1186/1475-2859-10-S1-S22 (PMC3231929; doi:10.1186/1475-2859-10-S1-S22)
Supplement: Additional file 9 — CRISPR gene activity during phage infection [file 1475-2859-10-S1-S22-S9.doc]

Additional file 9. CRISPR gene activity during phage infection.


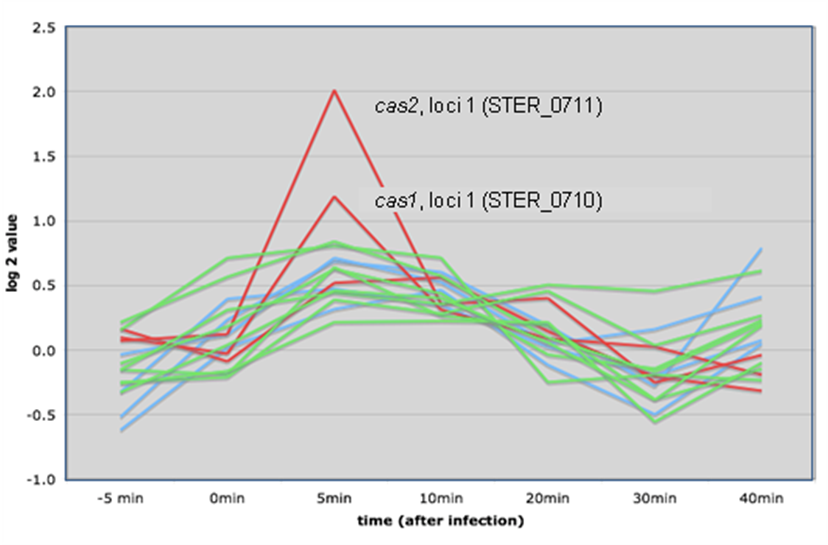


Each line indicates the change in expression of the 14 CRISPR-associated genes (*cas*) in the presence of phage DT1. Lines are color-coded based on the three CRISPR loci; red = CRISPR 1, green= CRISPR 2, blue = CRISPR 3. The log2 values indicate the fold change in gene expression, relative to the control, which was a parallel culture with no phage added.  Values were calculated as the log2 value of the ratio of treatment and control excitation values that were normalized among replicates. The two *cas* genes of which the expression was most differentially regulated are indicated.
